# A liposome–hydrogel composite ameliorates UVB-induced mouse skin photoaging through integrated antioxidant and extracellular matrix remodeling pathways

**DOI:** 10.1080/10717544.2026.2708405

**Published:** 2026-07-30

**Authors:** Yifan Liu, Kejia Chen, Jin Pei, Qi Song, Xinlu Wang, Chuhan Ma, Yao Li, Liangping Yu

**Affiliations:** a Department of Clinical Pharmacy, The First Hospital of Jilin University, Changchun, People's Republic of China; b Department of Biopharmacy, School of Pharmaceutical Sciences, Jilin University, Changchun, People's Republic of China

**Keywords:** Deer placental polypeptides, liposome, hydrogel, skin photoaging, antioxidant

## Abstract

Skin photoaging, predominantly caused by chronic ultraviolet B (UVB) exposure, is characterized by oxidative stress, collagen degradation, and disruption of the skin barrier. Deer placenta polypeptides (DPP) are rich in bioactive amino acids (AAs); however, their antioxidant and dermo-protective effects remain insufficiently elucidated, and their topical application is limited by enzymatic instability and poor transdermal permeability. To overcome these limitations, we developed a liposome–hydrogel composite delivery system to enhance the stability, skin penetration, and bioactivity of DPP. DPP obtained *via* enzymatic hydrolysis exhibited a favorable AA profile, free radical–scavenging activity, and a low molecular weight distribution (3–14 kDa). DPP-loaded liposomes (DPP-LIP) demonstrated high encapsulation efficiency, uniform nanosize, and effective preservation of bioactivity. Incorporation of DPP-LIP into a sodium alginate (SA) hydrogel yielded a composite formulation (DPP-LIP-SA) with sustained-release properties and a 2.7-fold enhancement in transdermal permeation. In a UVB-induced photoaging mouse model, topical administration of DPP-LIP-SA markedly alleviated oxidative stress, inflammatory responses, DNA damage, and extracellular matrix degradation. Mechanistically, DPP-LIP-SA treatment activated the Nrf2/HO-1 antioxidant pathway, inhibited TLR4/MyD88/NF-κB-mediated inflammatory signaling, reduced reactive oxygen species accumulation and lipid peroxidation, and restored extracellular matrix homeostasis by promoting collagen synthesis while suppressing MMP-mediated collagen degradation. Collectively, these findings identify DPP as a potent bioactive peptide resource with intrinsic antioxidant and reparative properties and demonstrate that integration of nanocarriers with hydrogel matrices substantially enhances dermal bioavailability. This composite delivery platform shows strong potential as a peptide-based topical strategy for preventing and treating skin photoaging.

## Graphical Abstract

**Figure:**
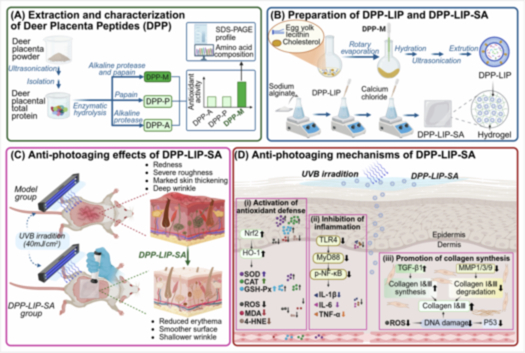

## 1. Introduction

Skin photoaging is a chronic degenerative process predominantly caused by prolonged exposure to ultraviolet (UV) radiation, including ultraviolet B (UVB, 290–320 nm) and ultraviolet A (UVA, 320–400 nm) (Tang et al. 2024a). It accounts for more than 80% of visible facial aging manifestations, such as wrinkle formation, epidermal barrier dysfunction, and pigmentation abnormalities (Leyane et al. 2022). The pathological basis of photoaging involves a self-perpetuating cycle characterised by excessive accumulation of reactive oxygen species (ROS), persistent inflammatory responses, and progressive degradation of the extracellular matrix (ECM), ultimately compromising dermal structural integrity (Zhang et al. 2024b; Tang et al. 2024b). UVB irradiation plays a central role in initiating this cycle by directly inducing DNA damage, promoting mitochondrial electron leakage, and triggering excessive ROS generation (Rahman et al. 2021; Kim et al. 2022). In parallel, UVA penetrates deeper into the dermis and activates membrane receptors on dermal fibroblasts (Ren et al. 2025), leading to the activation of nuclear factor kappa B (NF-κB) and mitogen-activated protein kinase (MAPK) signalling pathways. These cascades markedly upregulate matrix metalloproteinases (MMPs), thereby accelerating collagen degradation and elastic fibre disruption (Mo et al. 2022; Yao et al. 2022; Jang et al. 2023). Although topical antioxidants, such as vitamin C, and MMP inhibitors have been clinically employed to neutralise free radicals or attenuate collagen breakdown (Li et al. 2024), their therapeutic efficacy remains limited. Short biological half-lives (t_1/2_ < 6 h) (Wen et al. 2022), insufficient skin penetration, and an inability to simultaneously modulate multiple pathological pathways result in transient and suboptimal outcomes (Dosedel et al. 2021; Correia and Magina 2023). Collectively, these limitations underscore the urgent need for a skin-compatible therapeutic strategy capable of sustaining local drug concentrations while coordinating multi-pathway regulation.

Deer-derived bioactive substances, particularly peptides and growth-associated factors, have attracted increasing attention due to their capacity to promote fibroblast proliferation, tissue regeneration, and antioxidant defence (Shibagaki et al. 2024; Zhang et al. 2025). The placenta, a transient organ formed during mammalian gestation, is enriched with growth factors—including epidermal growth factor, fibroblast growth factor, and vascular endothelial growth factor—as well as ECM-modulatory signals that support dermal repair and regeneration (Biniazan et al., 2022; Salari et al., 2024). Consistent with these properties, placental extracts have been shown to upregulate ECM-related genes, such as collagen type I alpha 1 chain, elastin, and hyaluronan synthase 2, thereby enhancing collagen and elastin synthesis and contributing to improved dermal architecture (Shariatzadeh et al. 2021). Placental stromal cells promote the repair of radiation-damaged skin by enhancing hair follicle regeneration and reducing fibrosis, underscoring placenta-derived polypeptides (DPP) as a promising class of ECM-modulating and regenerative biomolecules for skin repair (Singh et al. 2025). Owing to their low molecular weight, DPP exhibit efficient cellular uptake and can modulate macrophage activity, scavenge reactive ROS, and promote keratinocyte migration (Wang et al. 2024a).

However, their susceptibility to enzymatic degradation and poor transdermal permeability (<15%) severely limit their bioavailability and therapeutic durability, making transdermal delivery the key barrier to clinical translation (Long et al. 2020).

Liposomes can encapsulate hydrophilic peptides to improve stability and tissue targeting (Cheng et al. 2021; Imraish et al. 2025), but suffer from burst release (>50% within 24 h) and limited storage stability (Maritim et al. 2021). Hydrogels enable sustained release and strong skin adhesion, yet their dense networks restrict drug diffusion and skin penetration (<20%) (Liang et al. 2021; Li et al. 2023; Cao et al. 2024). Integrating liposomes into hydrogels combines the strengths of both systems, improving formulation stability, enhancing transdermal transport, and minimising burst release (Zivari-Ghader et al. 2024; Xia et al. 2025; Yang et al. 2024a). Nevertheless, a delivery system capable of simultaneously stabilising DPP, sustaining its release, and enhancing skin penetration remains lacking. In this study, we developed an ionically crosslinked sodium alginate (SA) hydrogel encapsulating DPP-loaded liposomes (DPP-LIP-SA) to overcome the intrinsic instability and poor transdermal permeability of DPP. This system establishes a structure–function–delivery co-optimisation strategy for efficient DPP delivery and offers a promising platform for peptide-based anti-photoaging therapy.

## 2. Materials and methods

### 2.1. Materials

Deer placenta powder (20220926-LT-1) was obtained from Zhonglu Biotechnology Co., Ltd. (Changchun, China). Alkaline protease (S10154), papain (S10011), and vitamin C (Vc, A10012) were purchased from Shanghai Yuanye Bio-Technology Co., Ltd. (Shanghai, China). SA (S817374) was purchased from Shanghai Macklin Biochemical Technology Co., Ltd. (Shanghai, China). Egg phosphatidylcholine (MB5134) and cholesterol (MB6750) were purchased from Dalian Meilun Biological Technology Co., Ltd. (Dalian, China). Calcium chloride (Tocris-3148) and other analytical-grade reagents were obtained from Sinopharm Chemical Reagent Co., Ltd. (Shanghai, China).

### 2.2. Cell lines and animals

The NIH-3T3 mouse embryonic fibroblast cell line and HaCaT human keratinocyte cell line (Pricella, Wuhan, China) were cultured in Dulbecco’s modified Eagle’s medium (DMEM; Thermo Fisher Scientific, USA) supplemented with 8% foetal bovine serum (Gibco, USA) and 1% penicillin–streptomycin (Gibco, USA) and maintained at 37 °C in a humidified incubator with 5% CO_2_ (MCO-18AC, Panasonic, Japan). Female Kunming mice aged 6–8 weeks (20.0 ± 2.0 g) were purchased from Changsheng Biotechnology Co., Ltd. (Liaoning, China). Animals were kept in a specific pathogen-free grade animal centre with free access to food and water. All animal experiments were approved by the Committee on Animal Research and Ethics of the School of Pharmacy, Jilin University (No. 20240100), and were conducted in accordance with the NIH Guide for the Care and Use of Laboratory Animals. All animal studies were reported in compliance with the ARRIVE (Animal Research: Reporting of In Vivo Experiments) guidelines.

### 2.3. Extraction and quantification of DPTP and DPP

Deer placental total protein (DPTP) was extracted using an ultrasonication-assisted water extraction method. Subsequently, 5 g of deer placenta powder was dispersed in 500 mL distilled water (1:100, w/v), followed by magnetic stirring (30 min), ultrasonication (100 W, 2 h), centrifugation (5,000 × g, 10 min), and filtration (0.45 μm). The supernatant was lyophilised and quantified using the bicinchoninic acid (BCA; Beyotime, Shanghai, China) assay. The calibration curve is provided in Supplementary Fig. S1A. DPTP yield (%) = (protein mass in DPTP/mass of raw placenta powder) × 100%.

For enzymatic hydrolysis, DPTP (10 mg/mL) was incubated at 55 °C for 4 h with alkaline protease (pH 9.0, 4,000 U/g protein), papain (pH 7.0, 4,000 U/g protein), or a 1:1 (w/w) mixture of both enzymes (pH 7.5). Reactions were terminated by heating at 95 °C for 10 min, followed by centrifugation at 8,000 × g for 10 min. The supernatants were lyophilised to obtain DPP-A (alkaline hydrolysate), DPP-P (papain hydrolysate), and DPP-M (mixed hydrolysate). Peptide content of DPP was determined using the Folin–Ciocalteu method with tyrosine as the standard. The calibration curve is shown in Supplementary Fig. S1B. DPP yield (%) = (peptide mass in DPP/protein mass in DPTP) × 100%. DPP was extracted every three months between October 1, 2022 and January 1, 2025. After each extraction, the concentration of DPP was determined, and the samples were aliquoted and stored at −80 °C.

### 2.4. *In vitro* antioxidant activity of DPP

For the 2,2-diphenyl-1-picrylhydrazyl (DPPH) assay, samples (1.0–8.0 mg/mL) were mixed with 0.04% DPPH (B25609, Yuanye, Shanghai, China) ethanol solution (1:1, v/v), incubated in the dark for 30 min. Absorbance was measured at 517 nm using a UV–Vis spectrophotometer (UV-2800A, Unico, China). For the 2,2′-azino-bis(3-ethylbenzothiazoline-6-sulphonic acid) (ABTS) assay, samples (0.25–1.5 mg/mL) were mixed with a pre-oxidised ABTS stock solution (S19198, Yuanye, Shanghai, China). After incubation for 10 min, absorbance was measured at 734 nm. For hydroxyl radical scavenging assay, a reaction mixture containing FeSO_4_ (9.0 mM), salicylic acid-ethanol solution, and H_2_O_2_ (8.8 mM) was prepared at equal volumes (1 mL each). After incubation with sample at 37 °C for 15 min, absorbance was measured at 510 nm. The hydroxyl radical scavenging rate was calculated in the same way as ABTS. Ultrapure water served as the blank for all assays. Scavenging rates were calculated as:

$$\mathrm{DPPH scavenging}\;(\%)=\frac{A_{\mathrm{DPPH}+\mathrm{water}}-A_{\mathrm{DPPH}+\mathrm{sample}}+A_{\mathrm{ethanol}+\mathrm{sample}}}{A_{\mathrm{DPPH}+\mathrm{water}}}\times 100\%$$

$$\mathrm{ABTS or hydroxyl scavenging}\;(\%)=\frac{A_{\mathrm{blank}}-A_{\mathrm{sample}}}{A_{\mathrm{blank}}}\times 100\%$$

### 2.5. Molecular weight and amino acid (AA) composition of DPP

The molecular weight distribution of DPP was analysed using sodium dodecyl sulphate–polyacrylamide gel electrophoresis (SDS-PAGE). DPP samples (10 µg per lane) were mixed with 2 × SDS loading buffer, denatured at 95 ℃ for 5 min, centrifuged at 10,000 × g for 10 min, and loaded onto precast gels (3–20 kDa; PR1300, Solarbio, Beijing, China). Electrophoresis was performed at 30 V for 2 h followed by 100 V for 6 h. Gels were stained with Coomassie Brilliant Blue R-250 (6104-59-2, Solarbio, Beijing, China) for 1 h and destained in methanol–acetic acid solution until clear band visualisation.

AA composition was determined using an automated Amino Acid analyser (HT-1100, HiTech, Qingdao, China). Samples were hydrolysed and analysed with a lithium citrate mobile phase (0.24 mL/min), followed by post-column ninhydrin derivatization (0.12 mL/min, 115 °C). Absorbance was recorded at 570 nm for primary amines and 440 nm for proline/hydroxyproline. AA contents were quantified using standard calibration curves and expressed as mg per 100 mg of sample. All experiments related to the characterisation of DPP were conducted between October 15, 2022 and December 16, 2022.

### 2.6. Preparation of DPP-LIP and DPP-LIP-SA

DPP-loaded liposomes (DPP-LIP) were prepared using a thin-film hydration method based on the optimised formulation (Supplementary Fig. S2–S3 and Supplementary Table S1–S4). The optimal formulation was primarily selected according to encapsulation efficiency (EE). Egg phosphatidylcholine and cholesterol (6:1, w/w) were dissolved in anhydrous ethanol containing 1% Tween-20. The mixture was sonicated (100 W, 5 s) and evaporated (120 rpm) to form a thin lipid film. The lipid film was hydrated with a DPP solution (drug: lipid = 1: 15, w/w) for 1 h to obtain crude DPP-loaded liposomes. The resulting suspension was then sonicated at 300 W for 10 min in an ice bath and subsequently extruded five times through 450 and 220 nm polycarbonate membranes to obtain uniformly sized DPP-LIP.

To fabricate the liposomal–hydrogel composite, DPP-LIP was incorporated into SA. Specifically, 1 mL of DPP-LIP was mixed with 0.75% (w/v) SA solution, and the resulting mixture was subjected to gradient crosslinking using 13.5 mM CaCl_2_. Blank carriers (LIP-SA) were prepared using the same procedure but without DPP. DPP-LIP and DPP-LIP-SA were freshly prepared for use between December 18, 2023 and January 17, 2025.

### 2.7. Characterisation of DPP-LIP and DPP-LIP-SA

The morphology of DPP-LIP and DPP-LIP-SA was imaged using transmission electron microscopy (TEM; Tecnai G2, FEI, USA) and scanning electron microscopy (SEM; JSM-7900F, JEOL, Japan), respectively. For TEM observation, DPP-LIP samples were diluted with ultrapure water, deposited onto carbon-coated copper grids, negatively stained with 3% phosphotungstic acid, air-dried at 22–25 °C, and subsequently imaged using TEM. The particle size, polydispersity index (PDI), and zeta potential of DPP-LIP were measured using dynamic light scattering (DLS; Nano-ZS90, Malvern, UK). EE and drug loading (DL) of DPP-LIP were determined after removal of unencapsulated DPP by dialysis (molecular weight cut-off 8–14 kDa; YA1071, Solarbio, China). The peptide content was quantified using the Folin–Ciocalteu method, and EE and DL were calculated as:

$$\mathrm{EE}\;(\%)=\frac{\mathrm{Weight}\;\mathrm{of}\;\mathrm{DPP}\;\mathrm{in}\;\mathrm{liposome}}{\mathrm{Total}\;\mathrm{weight}\;\mathrm{of}\;\mathrm{drug}\;\mathrm{added}}\times 100\%$$

$$\mathrm{DL}\;(\%)=\frac{\mathrm{Weight}\;\mathrm{of}\;\mathrm{DPP}\;\mathrm{in}\;\mathrm{liposome}}{\mathrm{Weight}\;\mathrm{of}\;\mathrm{carrier}}\times 100\%$$

### 2.8. *In vitro* release and transdermal permeation

The *in vitro* release behaviour of DPP from different formulations (DPP, DPP-LIP, and DPP-LIP-SA) was evaluated using a dialysis method. Briefly, 1.0 mL of each sample was loaded into dialysis bags (molecular weight cut-off 8–14 kDa; YA1071, Solarbio, China) and dialysed against 50 mL PBS (37 °C, 100 rpm). At predetermined intervals (0, 1, 2, 6, 12, 24, and 48 h), 1.0 mL of the release medium was withdrawn and replaced with fresh PBS. The DPP concentration was determined using the Folin–Ciocalteu assay. Cumulative release rates were calculated as follows:

$$\mathrm{Cumulative release}\;(\%)=\frac{\mathrm{CnV}+\sum_{i=1}^{n-1}\mathrm{CiVi}}{w}\times 100\%$$

C_n_ = drug concentration at time *n*, V = total release medium volume (50.0 mL), V_i_ = sampling volume (1.0 mL), and w = total drug mass in liposomes.

Transdermal permeation was assessed using Franz diffusion instrument (RYJ-12B, Huanghai, Shanghai, China). Excised, depilated mouse skin (effective diffusion area 2.2 cm^2^) was mounted between the donor and receptor compartments (8.0 mL). Receptor compartments contained saline maintained at 37 °C under 600 rpm agitation. Formulations were applied to the skin surface, and samples (0.2 mL) were collected at 0–24 h (replenished with fresh medium). The permeated amount of DPP was quantified using the Folin–Ciocalteu assay, and cumulative transdermal amount per unit area (Q_n_) was calculated as:

$$Q_{n}\;(\%)=\frac{\mathrm{CnV}+\sum_{i=1}^{n-1}\mathrm{CiVi}}{A}\times 100\%$$

Q_n_ = cumulative permeation per unit area, C_n_ = drug concentration at time *n* (μg/mL), V = receptor volume (8.0 mL), V_i_ = sampling volume (0.2 mL), and A = effective diffusion area (cm^2^).

### 2.9. Stability of DPP-LIP and DPP-LIP-SA

The stability of DPP-LIP and DPP-LIP-SA was assessed under short-term and long-term storage conditions. For DPP-LIP, leakage and physical stability were evaluated by monitoring DPP retention, particle size, PDI, and zeta potential after storage at 4 and 25 °C for 0–24 h, and again after 30 days at 4 °C. For DPP-LIP-SA, samples stored at 4 and 25 °C were visually inspected and subjected to centrifugation (4,000 rpm, 30 min, 4 °C) at 1, 2, 6, 12, and 16 weeks to assess precipitation or phase separation. For content stability, three batches of DPP-LIP-SA stored at 4 °C were sampled monthly for 5 months, dispersed by vortexing and brief sonication, filtered (0.22 μm), and analysed for DPP concentration using the Folin–Ciocalteu assay. The pH values of each batch were measured monthly for 6 months to evaluate chemical stability during storage. All experiments related to the characterisation of DPP-LIP and DPP-LIP-SA were conducted between January 4, 2024 and May 26, 2024.

### 2.10. Cell treatment for *in vitro* assays

For *in vitro* experiments, NIH-3T3 and HaCaT cells were assigned to four groups. The *Normal* group was cultured in serum-free DMEM without UVB irradiation. The *Model* group was incubated with serum-free DMEM and exposed to UVB irradiation (wavelength 311 nm, TL20w/12RS, Philips, The Netherlands) for 5 min. The *DPP* group received DPP (150 µg/mL, prepared in serum-free DMEM) for 24 h followed by UVB irradiation for 5 min. The *DPP-LIP* group received DPP-LIP (equivalent to 150 µg/mL DPP, diluted in serum-free DMEM) for 24 h and was subsequently exposed to UVB irradiation for 5 min. The ultraviolet B (UVB) dose was set at 40 mJ/cm^2^ for all groups. All cell-based experiments were conducted between January 4, 2024 and August 6, 2024.

### 2.11. Cell viability assay

NIH-3T3 and HaCaT cells were seeded into 96-well plates (5 × 10^3^ cells/well) and cultured overnight. Cells were then treated with DPP or DPP-LIP at various DPP-equivalent concentrations (0–150 μg/mL) in serum-free DMEM. After 24 h of incubation at 37 °C, cell viability was measured using the Cell-Counting Kit 8 (CCK-8) assay, and absorbance at 450 nm was recorded using a microplate reader (DNM-9602, Beijing Pulang, China).

### 2.12. Wound scratch assay

NIH-3T3 were seeded into 6-well plates (2 × 10^5^ cells/well) and cultured to full confluence. A straight scratch was created using a sterile 200 μL pipette tip, and floating cells were removed by washing with PBS. Cells were then subjected to the corresponding treatments described in Section 2.10 (*Model*, *DPP*, and *DPP-LIP* groups), and incubated for 36 h. Images of the wound area were acquired at 0, 24, and 36 h using an inverted microscope. Migration rate (%) = (0 h scratch area - scratch area at each time point)/0 h scratch area × 100%.

### 2.13. Intracellular ROS detection

NIH-3T3 cells were seeded into 6-well plates (2 × 10^5^ cells/well) for 24 h, and then subjected to the treatments described in Section 2.10 (*Normal*, *Model*, *DPP*, and *DPP-LIP* groups). After treatment, cells were washed twice with PBS, followed by incubation with 2′,7′-dichlorodihydrofluorescein diacetate (DCFH-DA, 10 μM, S0033M, Beyotime, Shanghai, China) at 37 °C for 30 min in the dark. Excess probe was removed by an additional PBS wash. Intracellular ROS levels were imaged using a fluorescence microscopy (Ts2-FL, Nikon, Japan) and quantified using ImageJ software.

### 2.14. Apoptosis assay

NIH-3T3 cells were seeded into 6-well plates (2 × 10^5^ cells/well) for 24 h, and then subjected to the treatments described in Section 2.10 (*Normal*, *Model*, *DPP*, and *DPP-LIP* groups). After treatment, both floating and adherent cells were collected, washed twice with PBS, and resuspended in 1 × binding buffer (1 × 10^6^ cells/mL). The cell suspension (100 μL) was stained with 5 μL Annexin V–FITC and 5 μL PI (C1062L, Beyotime, Shanghai, China) for 15 min in the dark. After adding 600 μL binding buffer, samples were analysed using flow cytometry (Accuri C6 Plus, BD, USA), with 2 × 10^4^ cells recorded per sample. Apoptotic cells were quantified using FlowJo software. Apoptosis rate (%) = (early + late apoptosis)/total cells × 100%.

### 2.15 γ-H2AX immunofluorescence (IF) staining

HaCaT cells were seeded into 6-well plates (2 × 10^5^ cells/well) for 24 h, and then subjected to the treatments described in Section 2.10 (*Normal*, *Model*, *DPP*, and *DPP-LIP* groups). After fixation and blocking, the cells were incubated with an anti-*γ*-H2AX primary antibody overnight at 4 °C, followed by a fluorescence-conjugated secondary antibody. Nuclei were counterstained with DAPI. Fluorescence images were captured using a fluorescence microscope (XDS-1B, Optec, Chongqing, China) and quantified using ImageJ software.

### 2.16. ELISA and Western blot analysis

HaCaT cells were subjected to the treatments described in Section 2.10. Culture supernatants were collected and analysed for lactate dehydrogenase (LDH), matrix metalloproteinase-1 (MMP-1), MMP-3, and MMP-9 levels using commercial enzyme-linked immunosorbent assay (ELISA) kits (Jingmei Biological Technology Co., Ltd., Jiangsu, China) according to the manufacturers' instructions. For western blot analysis, total cellular proteins were extracted and analysed using the corresponding primary antibodies. Protein expression levels were quantified using ImageJ software.

### 2.17. *In vivo* effects and mechanisms of DPP-LIP-SA in UVB-induced skin photoaging mouse model

#### 2.17.1. Model establishment, grouping, and treatment procedures

A UVB-induced skin photoaging model was established by exposing mice to dorsal UVB irradiation (wavelength 311 nm, TL20w/12RS, Philips, The Netherlands) once every 2 days following a stepwise schedule: 10 min in week 1, 20 min in week 2, 30 min in weeks 3–4, and 45 min in weeks 5–8 at dose of 40 mJ/cm^2^. The dorsal skin (2 cm × 2 cm) was depilated regularly to ensure consistent UVB penetration. Female Kunming mice were randomly assigned into seven groups (*n* = 8): *Normal*, *Model*, *Vc*, *DPP*, *DPP-LIP*, *LIP-SA*, and *DPP-LIP-SA*. The *Normal* group received neither irradiation nor treatment. The *Model* group was subjected UVB irradiation without therapeutic intervention. For the treatment groups (*Vc*, *DPP*, *DPP-LIP*, *LIP-SA*, and *DPP-LIP-SA)*, the respective formulations were topically administered 30 min before each irradiation session. The formulations for all treatment groups were prepared at a concentration of 3.0 mg active ingredient/mL, and each formulation was applied topically at a dose of 1.0 mg active ingredient per cm^2^ of skin area. Skin surface features were photographed (Powershot 400 digital camera; Canon) under standardised lighting. Skin injury was evaluated using a standardised 0–5 grading system (Supplementary table S5) (Lee et al. 2021). Body weight was recorded throughout the experimental period.

At the end of the study, mice were anaesthetised with isoflurane and euthanized. Retro-orbital blood, full-thickness dorsal skin and major organs (heart, liver, spleen, lungs, kidneys) were collected for subsequent histological and biochemical analyses. The organ coefficients were calculated as: organ coefficient (%) = organ weight/body weight × 100%. Skin moisture content was determined by weighing fresh skin fragments (m_1_), drying them at 95 °C to constant weight (m_2_), and calculating as: skin moisture content (%) = (m_1_ − m_2_)/m_1_ × 100%. All animal experiments were conducted between August 12 2024 and January 17 2025.

#### 2.17.2. Histological and IF analyses of skin tissues

Paraffin-embedded dorsal skin tissues were sectioned and subjected to Hematoxylin and eosin (H&E), Masson’s trichrome, Sirius red, and IF staining. Histological alterations, collagen fibre organisation, and protein expression in skin tissues were evaluated using bright-field, polarised light, or fluorescence microscopy as appropriate. The contents of collagen I and collagen III and fluorescence intensities were quantified using ImageJ software.

#### 2.17.3. Oxidative stress– and inflammation-related biomarkers of skin tissues

Serum was obtained by centrifuging whole blood at 3,000 × g for 15 min at 4 °C, and used to measure superoxide dismutase (SOD), catalase (CAT), glutathione peroxidase (GSH-Px), malondialdehyde (MDA) using ELISA (Jingmei Biological Technology Co., Ltd., Jiangsu, China). Dorsal skin tissues (20–50 mg), previously snap-frozen in liquid nitrogen, were homogenised in radioimmunoprecipitation assay buffer containing phenylmethylsulfonyl fluoride and centrifuged at 12,000 × g (15 min, 4 °C). For each assay, 100 μL supernatants were analysed for SOD, CAT, GSH-Px, MDA, ROS, interleukin-1 beta (IL-1β), IL-6, and tumour necrosis factor-alpha (TNF-α) using ELISA.

#### 2.17.4. Western blot analysis of skin tissues

Proteins extracted from dorsal skin tissues were separated using SDS–PAGE, and transferred to polyvinylidene fluoride membranes. After blocking with 5% skim milk, the membranes were incubated with primary antibodies at 4 °C overnight, followed by horseradish peroxidase-conjugated secondary antibodies for 2 h at room temperature. Protein bands were visualised using enhanced chemiluminescence and quantified with the ImageJ software.

#### 2.17.5. RT-qPCR analysis of skin tissues

Total RNA was extracted from dorsal skin tissues using TRIzol reagent and reverse-transcribed into cDNA. Real-time quantitative PCR (RT-qPCR) was performed using a real-time PCR system (C6, TargetingOne, Beijing, China) to measure the expression of collagen I, collagen Ⅲ, MMP-3, TGF-β1, p53, and SOD2, with GAPDH as the internal reference. Primer sequences are listed in Supplementary Table S6.

### 2.18. Statistical analysis

Data are presented as the mean ± standard deviation (SD). Prior to parametric analysis, data normality and homogeneity of variance were assessed using the Shapiro–Wilk test and Brown–Forsythe/Bartlett’s tests, respectively. For normally distributed data with homogeneous variance, comparisons between two groups were performed using the Student’s *t*-test, whereas comparisons among multiple groups were analysed using one-way analysis of variance (ANOVA) followed by Tukey’s post hoc test. When these assumptions were not satisfied, appropriate non-parametric tests were applied. Analyses were performed using GraphPad Prism version 8.0. A *P*-values < 0.05 was considered statistically significant.

## 3. Results and discussion

### 3.1. Physicochemical characterisation and antioxidant activity of DPTP and DPP

**Figure 1.**
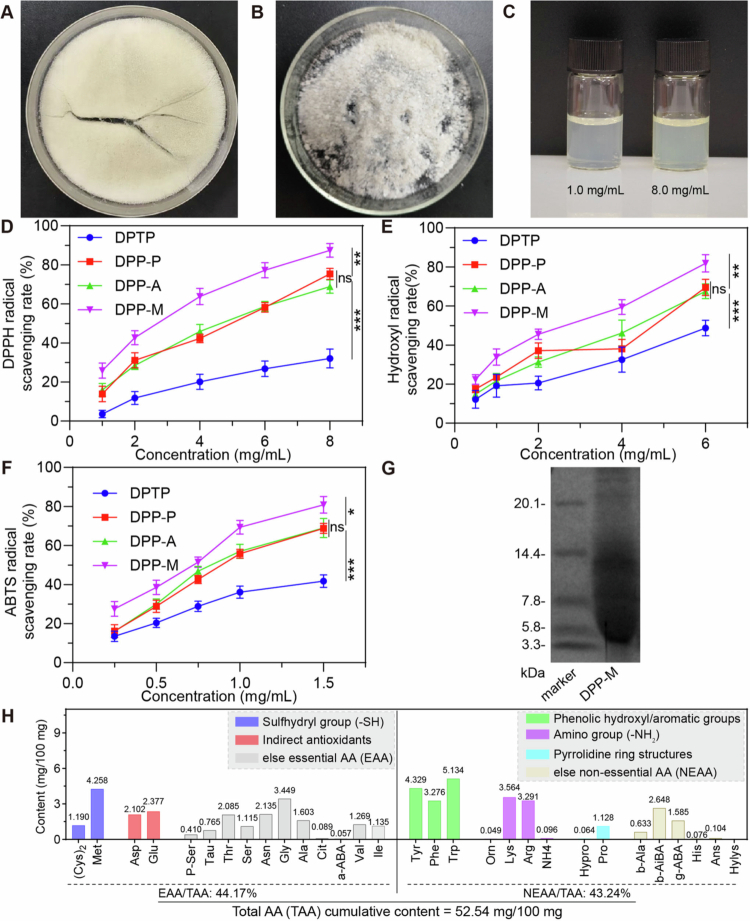
Physicochemical characterisation and antioxidant activity of deer placental total protein (DPTP) and deer placental peptides (DPP). (A) Appearance of lyophilised DPTP and (B) DPP powder. (C) Aqueous solutions of DPP at 1.0 and 8.0 mg/mL. (D) DPPH radical-scavenging activity of DPTP, DPP-P (papain hydrolysate), DPP-A (alkaline hydrolysate), and DPP-M (mixed hydrolysate). (E) Hydroxyl radical-scavenging activity. (F) ABTS radical-scavenging activity. (G) SDS-PAGE profiles of DPP-M. (H) Amino acid composition of DPP-M, grouped by functional categories. Data are presented as the mean ± SD, *n* = 3; *P < 0.05, **P < 0.01, ***P < 0.001.

DPTP were first extracted with a consistent yield of 56.80 ± 2.04% (Supplementary Fig. S1A) based on the BCA assay, indicating a reproducible and efficient extraction procedure. As shown in Figure 1A, the lyophilised DPTP appeared as a light-yellow spongy matrix with a uniform morphology, suitable for subsequent enzymatic hydrolysis. To obtain bioactive peptides, DPTP was hydrolysed using papain (DPP-*P*), alcalase (DPP-A), and a dual-enzyme mixture (DPP-M). As shown in Figure 1B and 1C, all DPP samples appeared as light-yellow powders and readily formed stable aqueous solutions. As detected by the Folin–Ciocalteu assay, DPP-M yielded significantly more peptides (38.23 ± 1.37%) than that of DPP-P (34.32 ± 1.09%) and DPP-A (33.64 ± 1.15%) (*P* < 0.05, Supplementary Fig. S1B).

To determine the antioxidant potential, DPTP and DPP samples were evaluated using DPPH, ABTS, and hydroxyl radical-scavenging assays *in vitro*. As shown in Figures 1D–F, all samples exhibited concentration-dependent scavenging activity. Among them, DPP-M exhibited superior antioxidant capacity with scavenging rates of 87.42 ± 3.58%, 80.95 ± 4.82%, and 81.87 ± 4.72% for DPPH, ABTS, and hydroxyl radicals, respectively. DPP-M was then chosen for subsequent experiments and hereafter referred to as DPP.

Peptides with molecular weights below 10 kDa are generally considered the primary contributors to antioxidant activity (Liu et al. 2023; Shekoohi et al. 2024; Zu et al. 2024; Mehmood et al. 2024). To further elucidate the molecular basis underlying its antioxidant activity, the molecular weight distribution of DPP was analysed by SDS-PAGE using a low-molecular-weight gel (3–20 kDa). As shown in Figure 1G, DPP was predominantly distributed between 3–14 kDa range, indicating successful enzymatic hydrolysis of deer placental proteins into small-molecular-weight peptides (Fang et al. 2021; Ma et al. 2021; Manassila et al. 2025).

Given that specific AA contributes directly to free radical scavenging, the composition and content of AAs in DPP were analysed. As shown in Figure 1H, DPP exhibited a balanced composition, with essential and non-essential AAs accounting for 44.17% and 43.24% of the total AA, respectively, indicating favourable nutritional characteristics (Wu 2013). Notably, 58.73% of AA in DPP possessed a well-defined antioxidant function. These included sulphur-containing, aromatic, basic, acidic, and pyrrolidine ring-containing amino acids, which are known to participate in free radical scavenging, ROS regulation, antioxidant enzyme activation, and metal ion chelation (Chiaverini and De Ley 2010; Wang et al. 2019; Yang et al. 2024; Chen et al. 2024; Ebrahimi et al. 2024; Zhou et al. 2024; Qin et al. 2024; Kumar et al. 2025). Collectively, the enrichment of these functional amino acids provides a structural basis for the antioxidant activity of DPP. Collectively, the enrichment of these functional AAs provides a structural basis for the antioxidant activity of DPP.

### 3.2. Characterisation and stability of DPP-LIP

**Figure 2.**
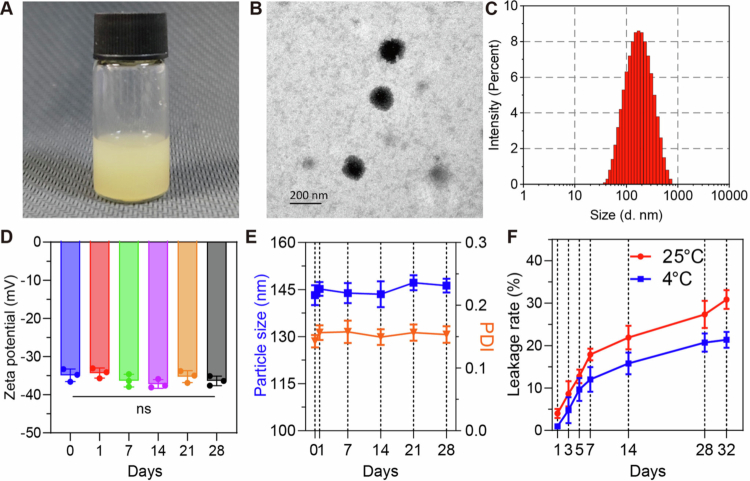
Physicochemical characterisation and storage stability of deer placental peptide-loaded liposomes (DPP-LIP). (A) Appearance of DPP-LIP. (B) Representative TEM images of DPP-LIP. Scale bar = 200 nm. (C) Particle size distribution of DPP-LIP. (D) Zeta potential changes of DPP-LIP during storage at 4 °C for 28 days. (E) Size and PDI changes of DPP-LIP during storage at 4 °C for 28 days. (F) Leakage rate of DPP-LIP during storage at 4 and 25 °C for 32 days.

To enhance the stability and facilitate transdermal delivery of DPP, the peptide was encapsulated into liposomes (DPP-LIP). Based on EE-guided single-factor and orthogonal optimisation analyses (Supplementary Fig. S2–S3 and Supplementary Table S1–S4), the optimal phospholipid-to-cholesterol ratio and drug-to-lipid ratio were determined to be 6:1 and 1:15, respectively. EE and DL were determined to be 77.45 ± 0.97% and 2.88 ± 0.14%, respectively (Table 1). As shown in Figure 2A, the freshly prepared DPP-LIP exhibited a uniform pale-yellow appearance without visible precipitation, aggregation, or phase separation, reflecting good colloidal stability.

**Table 1.** Characterisation of DPP-LIP (Mean ± SD, *n* = 3).

Formulation	Particle Size (nm)	PDI	Zeta Potential (mV)	EE (%)	DL (%)
DPP-LIP	145.1 ± 1.73	0.192 ± 0.028	−34.9 ± 0.536	77.45 ± 0.97	2.88 ± 0.14

As shown in Figure 2B, TEM images revealed spherical vesicles with uniform morphological integrity. As shown in Figure 2C and Table 1, DLS assay confirmed a hydrodynamic diameter of 145.1 ± 1.73 nm with a low PDI (0.192 ± 0.028), meeting the dimensional requirements for efficient percutaneous penetration while ensuring formulation homogeneity. The zeta potential was −34.9 ± 0.536 mV, indicating strong electrostatic stabilisation and contributing to colloidal stability during storage (Gnidovec et al. 2025).

The storage stability of DPP-LIP was subsequently evaluated. As shown in Figure 2D–E, particle size, PDI, and zeta potential remained essentially unchanged during 28 days of storage at 4 °C, indicating good physical stability under refrigerated conditions (Maritim et al. 2021). As shown in Figure 2F, drug leakage remained below 18% during 32 days of storage at 4 °C but exceeded 30% at 25 °C (Figure 2F). This temperature-dependent behaviour aligns with the thermal modulation of the lipid bilayer permeability and molecular diffusion rates (Maritim et al. 2021).

### 3.3. Protective effects of DPP-LIP against UVB-induced cellular injury and ECM degradation in NIH-3T3 and HaCaT cells

**Figure 3.**
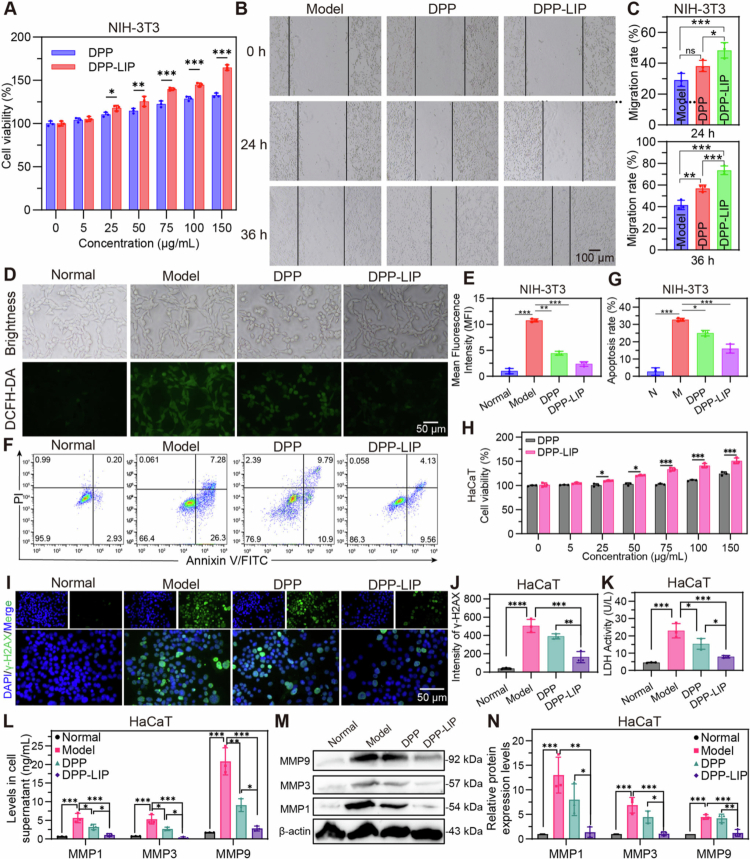
Protective effects of deer placental peptide (DPP)-loaded liposomes (DPP-LIP) on proliferation, migration, and ultraviolet B (UVB)-induced damage in NIH-3T3 and HaCaT cells. (A) Dose-dependent effects of DPP-LIP on NIH-3T3 cell proliferation measured using a CCK-8 assay. (B) Representative wound scratch images at 24 and 36 h after DPP or DPP-LIP treatment and UVB irradiation. Scale bar = 100 µm. (C) Quantitative analysis of the scratched area at 24 and 36 h. (D) Fluorescence microscopy images of intracellular ROS in NIH-3T3 cells detected using DCFH-DA probe. Scale bar = 50 µm. (E) Representative Annexin V/PIE staining and flow cytometry plots showing UVB-induced apoptosis and its attenuation by DPP or DPP-LIP pre-treatment. (F) ROS fluorescence intensity quantitated by FlowJo and GraphPad microsofts. (G) Quantitative analysis of the cell apoptosis rate. (H) Cell viability of HaCaT cells determined using a CCK-8 assay. (I) Representative immunofluorescence images of γ-H2AX in HaCaT cells for assessment of UVB-induced DNA damage. Scale bar = 50 µm. (J) Quantitative analysis of γ-H2AX fluorescence intensity using ImageJ software. (K) Lactate dehydrogenase (LDH) release levels in HaCaT cell supernatants using ELISA. (L) ELISA analysis of MMP-1, MMP-3, and MMP-9 levels in HaCaT cell supernatants. (M) Representative Western blot bands of MMP-1, MMP-3, and MMP-9 in HaCaT cells. (N) Quantitative analysis of MMP-1, MMP-3, and MMP-9 protein expression levels using ImageJ software. Data are presented as the mean ± SD, *n* = 3; *P < 0.05, **P < 0.01, and ***P < 0.001.

Because fibroblast proliferation and migration are crucial for wound repair and tissue regeneration, the proliferative effects of DPP and DPP-LIP on NIH-3T3 cells were first assessed using CCK-8 and wound scratch assays (Liang et al. 2021). As shown in Figure 3A, both formulations exhibited a dose-dependent enhancement of cell viability (0–150 µg/mL), whereas DPP-LIP demonstrated significantly greater efficacy than free DPP in the 25–150 µg/mL range. As illustrated in Figures 3B–C, DPP-LIP significantly accelerated wound healing, reaching 48% *vs.* 38% of DPP at 24 h and 73% *vs.* 56% of DPP at 36 h.

Because excessive ROS accumulation is a key driver of UVB-induced oxidative injury and apoptosis during skin photoaging (Kim et al. 2022), intracellular ROS levels and apoptotic cell death were subsequently assessed in NIH-3T3 fibroblasts. As shown in Figure 3D, UVB irradiation induced significant ROS accumulation, whereas DPP and DPP-LIP significantly reduced ROS generation, with DPP-LIP exhibiting superior ROS scavenging activity (Figure 3E). Consistently, Annexin V/PIE analysis demonstrated that UVB increased apoptosis from 2.66% in the normal group to 32.68% in the UVB group. DPP pre-treatment reduced the apoptosis rate to 24.91%, while DPP-LIP further reduced it to 15.96%, indicating that liposomal encapsulation enhanced DPP anti-apoptotic capacity (Figure 3F–G).

Following the evaluation of fibroblast proliferation, migration, and oxidative stress-related repair activity in NIH-3T3 cells, HaCaT keratinocytes were further employed as a more skin-relevant UVB-induced photoaging model to evaluate epidermal cellular injury, DNA damage, and ECM degradation. As shown in Figure 3H and 3K, UVB irradiation reduced cell viability and increased LDH release compared with the normal group, indicating pronounced cellular injury. Whereas both DPP and DPP-LIP alleviated UVB-induced cytotoxicity, and DPP-LIP exhibited stronger protective effects than did free DPP.

Because DNA damage is a hallmark of UVB-induced skin photoaging, *γ*-H2AX IF staining was performed to assess DNA damage in HaCaT cells. As shown in Figure 3I–J, UVB irradiation markedly increased *γ*-H2AX fluorescence intensity, whereas both DPP and DPP-LIP significantly attenuated *γ*-H2AX accumulation, with DPP-LIP showing superior protective efficacy.

Because matrix metalloproteinases are key mediators of ECM degradation during skin photoaging, MMP expression was further evaluated in HaCaT cells. As shown in Figure 3L, UVB irradiation increased the secretion of MMP-1, MMP-3, and MMP-9 in cell supernatants, whereas DPP-LIP markedly suppressed their release. Consistently, Western blot analysis demonstrated that DPP-LIP downregulated the protein expression of MMP-1, MMP-3, and MMP-9 compared with that of the UVB model group (Figure 3M–N and Supplementary Fig. S4). Collectively, these findings demonstrate that DPP-LIP effectively protects keratinocytes against UVB-induced cellular injury, DNA damage, and ECM degradation.

### 3.4. Development, controlled release, and stability of DPP-LIP-SA

**Figure 4.**
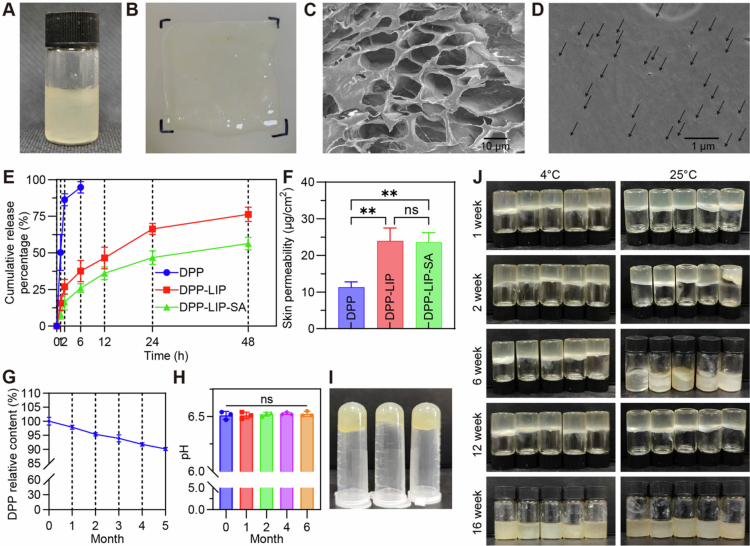
Characterisation, release behaviour, and stability of sodium alginate-based deer placental peptide-loaded liposomes (DPP-LIP-SA). (A) Appearance of DPP-LIP-SA. (B) Topical application of DPP-LIP-SA. (C, D) SEM images showing the internal porous structure of DPP-LIP-SA. Scale bar = 10 or 1 μm. (E) Cumulative release rates of DPP, DPP-LIP, and DPP-LIP-SA within 48 h. (F) Cumulative skin permeation of DPP, DPP-LIP, and DPP-LIP-SA per unit area over 24 h. (G) Relative content of DPP-LIP-SA during a 5-month storage at 4 °C. (H) pH stability of DPP-LIP-SA within 6 months. (I) Centrifugal stability of DPP-LIP-SA. (J) Visual appearance alterations of DPP-LIP-SA at 4 and 25 °C storage conditions over 16 weeks (*n* = 5). Data are presented as the mean ± SD, *n* = 3; **P < 0.01.

To enhance the topical delivery and sustained release of DPP, we developed a liposome–hydrogel composite, DPP-LIP-SA. As shown in Figure 4A, the formulation appeared as a homogeneous pale yellow semi-solid gel without visible particles or phase separation, demonstrating a uniform internal structure and good physical stability. As shown in Figure 4B, DPP-LIP-SA formed a uniform coating when spread on glass, indicating its suitability for topical applications (Kim et al. 2022). SEM images (Figure 4C) further revealed a porous network, providing a 3D diffusion matrix for prolonged release. At higher magnification (Figure 4D), uniformly distributed submicron spherical structures were observed, consistent with the embedded liposomal vesicles.

The release behaviour of DPP-LIP-SA was subsequently evaluated *in vitro*. As shown in Figure 4E, free DPP exhibited rapid diffusion, with almost complete release within 6 h. In contrast, DPP-LIP and DPP-LIP-SA exhibited sustained release, reaching 76.31% and 56.39% at 48 h, respectively. Among them, DPP-LIP-SA exhibited the strongest sustained-release effect. Transdermal permeation experiments (Figure 4F) further demonstrated that both DPP-LIP (23.93 μg/cm^2^) and DPP-LIP-SA (23.60 μg/cm^2^) achieved significantly higher cumulative skin permeation within 24 h than that of free DPP (11.33 μg/cm^2^). Notably, DPP-LIP-SA exhibited a comparable permeation level to DPP-LIP, indicating that the incorporation of the hydrogel network successfully provided sustained release without hindering transdermal delivery.

The storage stability of DPP-LIP-SA was further assessed. As shown in Figure 4G, DPP-LIP-SA maintained 90.17% of its drug content after 5 months of storage at 4 °C. In addition, the formulation maintained a stable pH of 6.50 ± 0.02 (Figure 4H), and showed no visible precipitation or phase separation after centrifugation (Figure 4I). Stability testing demonstrated that DPP-LIP-SA remained structurally stable at 4 °C for at least 16 weeks, whereas progressive destabilization was observed during storage at 25 °C (Figure 4J). Together, these results demonstrated that DPP-LIP-SA provides effective encapsulation, sustained release, high transdermal permeation, and robust storage stability, supporting its potential as a safe and effective topical delivery system (Yin et al. 2007; Luo et al. 2023; Kaur et al. 2024).

### 3.5. DPP-LIP-SA attenuates UVB-induced skin injury in mice

**Figure 5.**
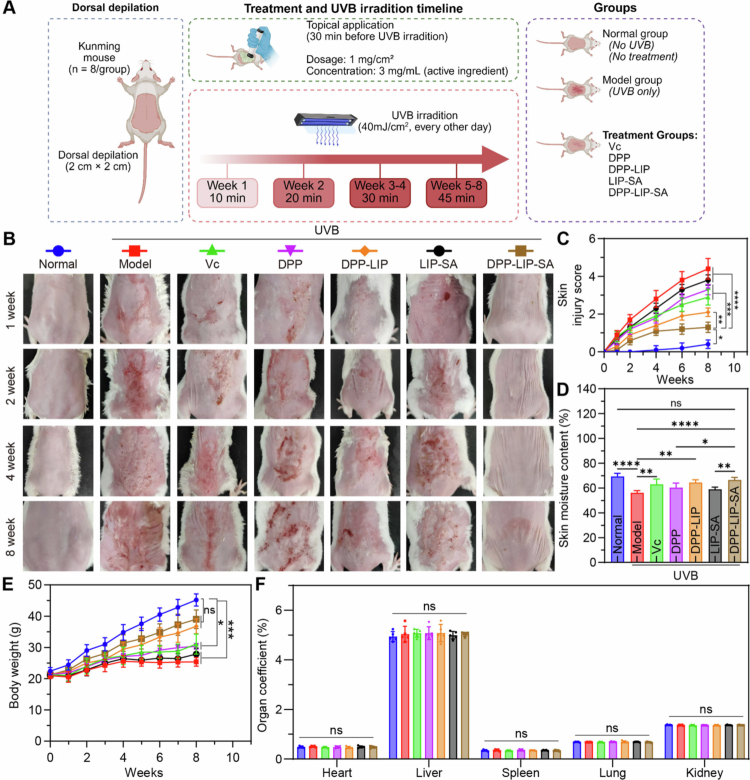
Therapeutic effects of different formulations in an ultraviolet B (UVB)-induced mouse skin injury model. (A) Schematic overview of UVB irradiation and topical administration protocol. (B) Photographs of UVB-irradiated mouse dorsal skin after different treatments. (C) Skin injury scores in different treatment groups. (D) Moisture content of dorsal skin in different treatment groups. (E) Body weight alterations in different treatment groups. (F) Organ coefficients of major organs after various treatments. Data are presented as the mean ± SD, *n* = 8; *P < 0.05, **P < 0.01, ***P < 0.001, and ****P < 0.0001.

To evaluate the *in vivo* protective effects of DPP-LIP-SA on the skin, a UVB-induced photoaging mouse model was established (Figure 5A). After 8 weeks of UVB irradiation, the model group developed typical photoaging features, including erythema, epidermal thickening, dermal matrix degradation, and prominent wrinkles. As shown in Figure 5B, DPP-LIP-SA treatment effectively preserved skin appearance, exhibiting reduced erythema, smoother surface texture, and shallower wrinkle compared with other treatments. DPP-LIP offered moderate dermal protection, whereas free DPP and Vc demonstrated only modest improvements, and LIP-SA exhibited negligible protective effects. These observations were consistent with the injury scores (Supplementary table S5). As shown in Figure 5C, DPP-LIP-SA treatment significantly reduced skin injury scores compared with all other UVB-treated groups (*P* < 0.01). Because skin moisture content is a key indicator of barrier integrity (Cao et al. 2024), skin moisture content was further assessed. As shown in Figure 5D, UVB irradiation caused severe moisture loss in the model group, whereas DPP-LIP-SA treatment restored skin moisture content to normal levels (*P* > 0.05) and surpassed all other interventions (*P* < 0.05), demonstrating effective protection against UVB-induced moisture loss and enhancement of skin barrier function.

To assess systemic safety, the body weight and organ coefficients were monitored. As shown in Figure 5E, UVB irradiation slowed weight gain in the model group, whereas DPP-LIP-SA treatment restored growth to levels similar to those in the normal controls. As shown in Figure 5F, organ coefficient analysis revealed no significant differences between the treatment and normal groups (*P* > 0.05), indicating that topical administration did not induce systemic toxicity. These results indicate that DPP-LIP-SA treatment effectively protects the skin against UVB-induced damage, enhances moisture and barrier function, and exhibits a favourable safety profile.

### 3.6. Histopathological analysis of cutaneous tissues

**Figure 6.**
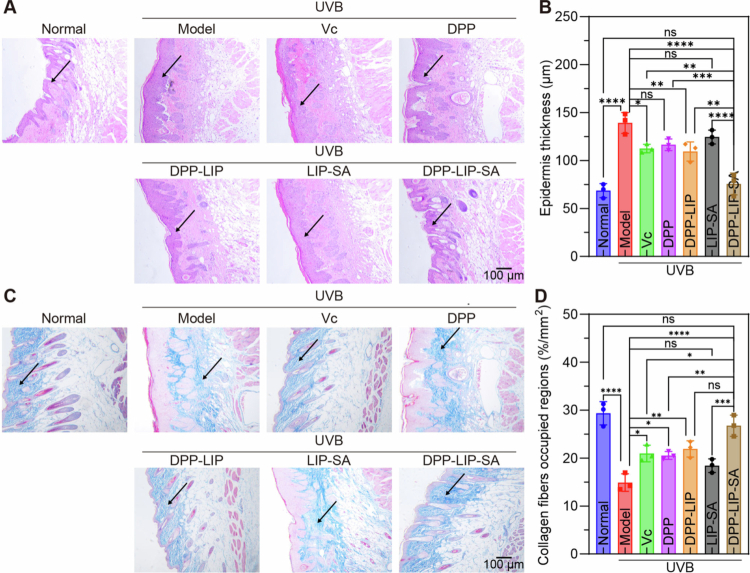
Effects of different treatments on histopathology in ultraviolet B (UVB)-irradiated mouse skin. (A) H&E-stained sections of the dorsal skin (scale bar = 100 μm; arrows indicate epidermal thickness); (B) Quantitative analysis of epidermal thickness based on H&E staining; (C) Masson’s trichrome–stained sections of skin tissue (scale bar = 100 μm, arrows indicate collagen fibres); (D) Quantitative analysis of collagen content based on Masson staining. Data are presented as the mean ± SD, *n* = 3; *P < 0.05, **P < 0.01, ***P < 0.001, and ****P < 0.0001.

To assess the UVB-induced skin architecture damage, histopathological analysis was performed using H&E and Masson staining. As shown in Figure 6A, the normal group displayed intact epidermal architecture with uniform thickness. In contrast, the model group exhibited typical photoaging features, including epidermal acanthosis, hyperkeratosis, and fragmentation of dermal collagen fibres. Among the treatment groups, Vc partially reduced epidermal thickening but failed to improve collagen organisation. DPP normalised epidermal thickness but provided limited dermal repair. DPP-LIP significantly enhanced collagen alignment and epidermal integrity, whereas the DPP-LIP-SA group restored both epidermal and dermal architecture to a near-normal skin state.

Masson staining further substantiated these observations. As shown in Figure 6C, normal skin exhibited dense, continuous collagen networks, whereas the model group showed disrupted, sparsely distributed collagen fibres. DPP-LIP-SA treatment effectively restored collagen distribution, fibre diameter, and alignment to normal levels, outperforming all other treatments. Quantitative analyses of the epidermal thickness (Figure 6B) and collagen density (Figure 6D) confirmed the histological observations, demonstrating the superior protective capacity of DPP-LIP-SA in UVB-induced skin damage.

### 3.7. DPP-LIP-SA attenuates UVB-induced oxidative stress and activates the endogenous antioxidant system in mouse skin

**Figure 7.**
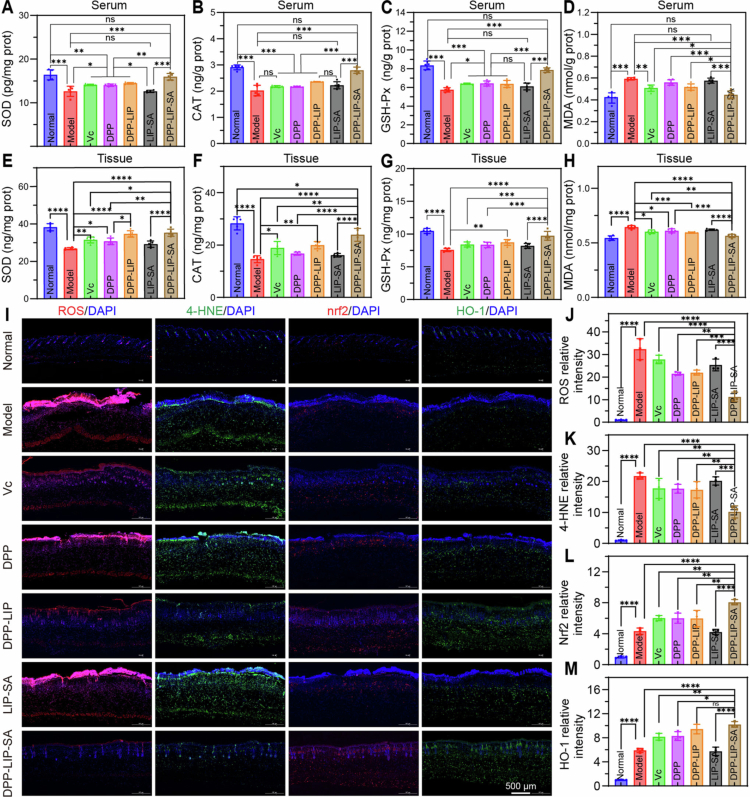
Sodium alginate-based deer placental peptide-loaded liposomes (DPP-LIP-SA) attenuate ultraviolet B (UVB)-induced oxidative stress. (A–D) ELISA analysis of serum oxidative stress biomarkers, including (A) superoxide dismutase (SOD), (B) catalase (CAT), (C) glutathione peroxidase (GSH-Px) activities, and (D) malondialdehyde (MDA) content. (E–H) ELISA analysis of oxidative stress biomarkers in skin tissues, including (E) SOD, (F) CAT, and (G) GSH-Px activities, and (H) MDA content. (I) Representative fluorescence images of ROS, 4-Hydroxynonenal (4-HNE), Nrf2, and HO-1 in skin tissues. (J–M) Quantitative analysis of (J) ROS, (K) 4-HNE, (L) Nrf2, and (M) HO-1 fluorescence intensities. Data are presented as the mean ± SD, *n* = 5 for ELISA and *n* = 3 for fluorescence analysis; *P < 0.05, **P < 0.01, ***P < 0.001, and ****P < 0.0001.

To investigate the antioxidant effects of DPP-LIP-SA *in vivo*, oxidative stress-related indicators were evaluated in both serum and skin tissue. As shown in Figure 7A–D, UVB irradiation significantly suppressed the activity of antioxidant enzymes, such as SOD, CAT, and GSH-Px, and elevated MDA in serum, indicating pronounced systemic oxidative damage. Similar results were observed in skin tissues (Figure 7E–H), demonstrating severe skin oxidative injury. DPP-LIP-SA treatment strongly increased antioxidant enzyme activities and reduced MDA levels (*P* < 0.001 *vs.* Model group). Notably, this effect was significantly greater than that of free DPP, highlighting the contribution of the liposomal–hydrogel delivery system in enhancing the antioxidant efficacy.

As shown in Figure 7I–J and Supplementary Fig. S5A, both ELISA and fluorescence staining analyses demonstrated that UVB irradiation caused marked ROS accumulation in skin tissues, whereas DPP-LIP-SA treatment significantly decreased ROS levels (*P* < 0.001 *vs.* Model group). In addition, fluorescence staining showed that UVB exposure markedly increased 4-Hydroxynonenal (4-HNE) accumulation in skin tissues, whereas DPP-LIP-SA treatment significantly decreased lipid peroxidation-associated fluorescence signals (Figure 7I and K).

To further evaluate endogenous antioxidant defences of DPP-LIP-SA, the expression of Nrf2, HO-1, and SOD2 was assessed. As shown in Figure 7I and 7L–M, UVB irradiation markedly weakened the fluorescence intensity of Nrf2 and HO-1, and reduced SOD2 mRNA expression compared with that of Normal group (Supplementary Fig. S5B), indicating impairment of the endogenous antioxidant system. In contrast, DPP-LIP-SA treatment significantly enhanced Nrf2 and HO-1 fluorescence signals, and increased SOD2 mRNA expression (*P* < 0.0001 *vs.* Model group), indicating the activation of endogenous antioxidant defences pathways (Tang et al. 2026). Collectively, these findings indicate that DPP-LIP-SA treatment effectively alleviates UVB-induced oxidative stress by suppressing ROS accumulation and lipid peroxidation while enhancing endogenous antioxidant capacity in skin tissues.

### 3.8. DPP-LIP-SA mitigates cutaneous inflammation by suppressing the TLR4/MyD88/NF-κB signalling pathway

**Figure 8.**
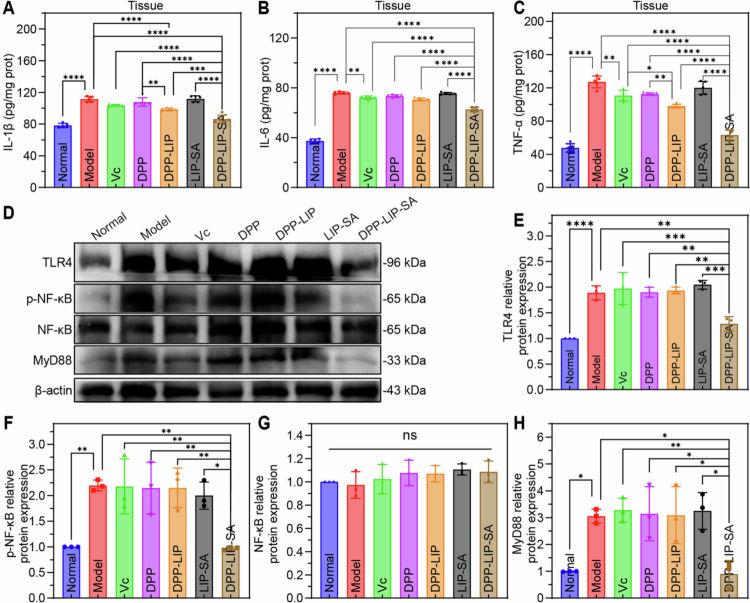
Sodium alginate-based deer placental peptide-loaded liposomes (DPP-LIP-SA) attenuate ultraviolet B (UVB)-induced inflammation. (A–C) ELISA analysis of (A) interleukin-1β (IL-1β), (B) interleukin-6 (IL-6), and (C) tumour necrosis factor-α (TNF-α) levels in mouse skin tissues. (D) Representative western blot bands of TLR4, MyD88, p-NF-κB, and NF-κB in skin tissues. (E–H) Quantitative analysis of (E) TLR4, (F) MyD88, (G) p-NF-κB, (H) and NF-κB protein expression levels. Data are presented as the mean ± SD, *n* = 5 for ELISA and *n* = 3 for western blot; *P < 0.05, **P < 0.01, ***P < 0.001, and ****P < 0.0001.

UVB-induced oxidative stress triggers a robust inflammatory response in the skin, which in turn exacerbates oxidative damage and reinforces a vicious cycle of photoaging (Lee et al. 2021). To determine whether DPP-LIP-SA can interrupt this cycle, we assessed its effects on pro-inflammatory cytokines. As shown in Figure 8A–C, UVB irradiation significantly increased IL-1β, IL-6, and TNF-*α* levels in skin tissues (*P* < 0.001 *vs. Normal* group). Treatment with DPP-LIP-SA significantly reduced the release of all three cytokines, outperforming Vc and the other formulations (*P* < 0.0001), demonstrating strong anti-inflammatory efficacy.

Because TLR4/MyD88/NF-κB signalling is closely associated with UVB-induced inflammatory responses during skin photoaging, the expression of related proteins was further evaluated using Western blot (Tang et al. 2026). As shown in Figure 8D–H and Supplementary Fig. S6, UVB irradiation markedly increased expression of TLR4, MyD88, and p-NF-κB compared that of the normal group. In contrast, DPP-LIP-SA treatment significantly suppressed the expression of above proteins, indicating that DPP-LIP-SA treatment effectively alleviates UVB-induced cutaneous inflammation, potentially through suppression of the TLR4/MyD88/NF-κB signalling pathway and subsequent decreasing of pro-inflammatory cytokine production.

### 3.9. DPP-LIP-SA facilitates ECM remodelling by maintaining the synthesis-degradation balance

**Figure 9.**
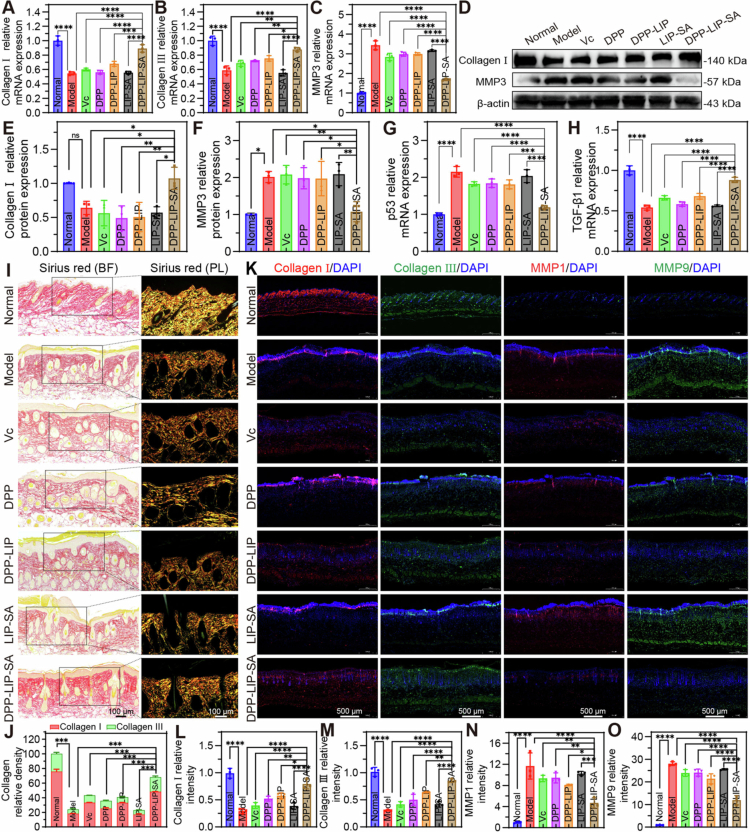
Sodium alginate-based deer placental peptide-loaded liposomes (DPP-LIP-SA) facilitate extracellular matrix (ECM) remodelling by coordinately regulating synthesis and degradation factors. (A–C) mRNA expression level of (A) collagen I, (B) collagen III, and (C) matrix metalloproteinase-3 (MMP-3) in skin tissues detected by qRT-PCR. (D) Representative western blot bands of collagen I and MMP-3 protein expression levels in skin tissues. (E–F) Quantitative analysis of (E) collagen I and (F) MMP-3 protein expression levels. (G–H) qRT-PCR analysis of (G) p53 and (H) transforming growth factor-β (TGF-β) mRNA expression levels in skin tissues. (I) Representative Sirius red staining images under bright-field (BF) and polarised light (PL) microscopy showing collagen fibre in skin tissues. Scale bar = 100 μm. (J) Quantitative analysis of collagen I and collagen III contents based on Sirius red staining under PL microscopy. (K) Representative immunofluorescence images of collagen I, collagen III, MMP-1, and MMP-9 in skin tissues. Scale bar = 500 μm. (L–O) Quantitative analysis of (L) collagen I, (M) collagen III, (N) MMP-1, and (O) MMP-9 fluorescence intensities. Data are presented as the mean ± SD, *n* = 3; *P < 0.05, **P < 0.01, ***P < 0.001, and ****P < 0.0001.

The disruption of dermal ECM homoeostasis is a hallmark of UVB-induced skin photoaging. Because ECM homoeostasis depends on the balance between collagen synthesis and matrix degradation mediated by matrix metalloproteinases (Niland et al. 2022; De Almeida et al. 2022; Lu et al. 2024; Wang et al. 2024), the effects of DPP-LIP-SA on ECM remodelling were further evaluated. As shown in Figure 9A–F and Supplementary Fig. S6, UVB irradiation significantly downregulated collagen I and collagen III expression, while markedly upregulating MMP-3, consistent with accelerated ECM breakdown. In contrast, DPP-LIP-SA treatment significantly enhanced collagen expression, while downregulating MMP-3, thereby re-establishing the synthesis–degradation equilibrium. These regulatory effects were stronger than that of free DPP, suggesting that the sustained-release liposomal–hydrogel delivery system for effective ECM remodelling (Serra 2024; Nieuwstraten et al. 2025).

Histological evaluation by Sirius red staining further confirmed the protective effects of DPP-LIP-SA on dermal collagen architecture. As shown in Figure 9I–J, UVB irradiation resulted in obvious collagen loss and disorganisation, accompanied by a marked reduction in both collagen I and collagen III contents under polarised light microscopy. In contrast, DPP-LIP-SA treatment significantly improved collagen deposition and restored collagen fibre organisation, indicating attenuation of UVB-induced collagen degradation (*P* < 0.001 *vs.* Model group). Furthermore, IF staining revealed that UVB exposure markedly decreased collagen I and collagen III fluorescence signals while increasing MMP-1 and MMP-9 expression in skin tissues (Figure 9K–O). DPP-LIP-SA treatment significantly reversed these changes, as evidenced by enhanced collagen I and collagen III fluorescence intensities and reduced MMP-1 and MMP-9 levels compared with that of the Model group (*P* < 0.001).

Upregulation of TGF-β1 mRNA (Figure 9G), a central regulator of collagen biosynthesis (Jiang et al. 2022), may contribute to the promotive effects of DPP-LIP-SA on matrix repair. Additionally, DPP-LIP-SA treatment significantly downregulated p53 mRNA (Figure 9H), suggesting an alleviation of UVB-induced cellular stress and DNA damage (Von Jan et al. 2024; Haddad et al. 2025). In summary, these findings demonstrate that DPP-LIP-SA treatment effectively attenuates UVB-induced ECM disruption by promoting collagen synthesis and suppressing matrix degradation, thereby preserving dermal collagen homoeostasis and improving skin architecture.

## 4. Conclusion

In this study, DPP with potent antioxidant activity was successfully prepared and incorporated into a liposomal–hydrogel delivery system (DPP-LIP-SA) to improve its stability, sustained release behaviour, and transdermal delivery efficiency. The optimised DPP-LIP-SA formulation exhibited favourable physicochemical properties, prolonged release characteristics, high skin permeation efficiency, and robust storage stability.

Both *in vitro* and *in vivo* experiments demonstrated that DPP-LIP-SA treatment alleviated UVB–induced skin photoaging. Mechanistically, DPP-LIP-SA treatment reduced ROS accumulation and lipid peroxidation, enhanced endogenous antioxidant defences through activation of the Nrf2/HO-1 pathway, suppressed UVB-induced inflammatory responses via inhibition of the TLR4/MyD88/NF-κB signalling pathway, and restored ECM homoeostasis by upregulating collagen synthesis while inhibiting MMP-mediated matrix degradation. These coordinated effects collectively contributed to the attenuation of epidermal injury, dermal collagen disruption, and skin barrier dysfunction induced by UVB irradiation.

Overall, these results establish an effective nanocarrier–hydrogel integrated delivery strategy for bioactive peptides, and highlight the therapeutic potential of DPP as a peptide-based topical intervention for skin photoaging. Nevertheless, although multiple signalling pathways associated with oxidative stress, inflammation, and ECM remodelling were identified, the direct molecular interactions and upstream regulatory mechanisms remain to be elucidated. Future studies focusing on pathway-specific modulation and molecular targeting may help clarify the involved mechanisms.

## Supplementary Material

Supplementary Material Supplementary Material

Supplementary Material.docx

## Data Availability

The data that support the findings of this research are available from the corresponding author, L.Y., upon reasonable request.
