# Supplementary material for: A liposome–hydrogel composite ameliorates UVB-induced mouse skin photoaging through integrated antioxidant and extracellular matrix remodeling pathways
Source: Drug Deliv. 2026 Jul 30;33(1):2708405. doi: 10.1080/10717544.2026.2708405 (PMC13425532; doi:10.1080/10717544.2026.2708405)
Supplement: Supplementary Material [file IDRD_A_2708405_SM5033.pdf]

NOTE: Please save this file locally before filling in the table, DO NOT work on the file within your internet browser as changes will not be saved. Adobe Acrobat Reader (available free here) is recommended for completion.

The ARRIVE guidelines 2.0: author checklist

The ARRIVE Essential 10

These items are the basic minimum to include in a manuscript. Without this information, readers and reviewers cannot assess the reliability of the findings.

Item		Recommendation	Section/line number, or reason for not reporting
**Study design**	1	For each experiment, provide brief details of study design including:	
		a. The groups being compared, including control groups. If no control group has been used, the rationale should be stated.	Normal, Model, Vc, DPP, DPP-LIP, LIP-SA, and DPP-LIP-SA.<br>The Normal group received neither UVB irradiation nor treatment. The Model group received UVB irradiation without therapeutic intervention. Treatment groups received topical formulations prior to UVB exposure.
		b. The experimental unit (e.g. a single animal, litter, or cage of animals).	The experimental unit was a single mouse.
**Sample size**	2	a. Specify the exact number of experimental units allocated to each group, and the total number in each experiment. Also indicate the total number of animals used.	Female Kunming mice were randomly assigned to seven groups with eight mice per group (n = 8), resulting in a total of 56 mice.
		b. Explain how the sample size was decided. Provide details of any *a priori* sample size calculation, if done.	The sample size was determined based on previous similar studies and practical considerations to ensure sufficient statistical power while minimizing animal use.
**Inclusion and exclusion criteria**	3	a. Describe any criteria used for including and excluding animals (or experimental units) during the experiment, and data points during the analysis. Specify if these criteria were established *a priori*. If no criteria were set, state this explicitly.	No a priori inclusion or exclusion criteria were defined.
		b. For each experimental group, report any animals, experimental units or data points not included in the analysis and explain why. If there were no exclusions, state so.	All animals enrolled in the study completed the experimental procedures and were included in the final analysis.
		c. For each analysis, report the exact value of *n* in each experimental group.	No animals or data points were excluded.
**Randomisation**	4	a. State whether randomisation was used to allocate experimental units to control and treatment groups. If done, provide the method used to generate the randomisation sequence.	Mice were randomly assigned to experimental groups using a simple randomization method.
		b. Describe the strategy used to minimise potential confounders such as the order of treatments and measurements, or animal/cage location. If confounders were not controlled, state this explicitly.	All animals were housed under identical environmental conditions, and treatments and measurements were conducted according to the same schedule to minimize potential confounding factors.
**Blinding**	5	Describe who was aware of the group allocation at the different stages of the experiment (during the allocation, the conduct of the experiment, the outcome assessment, and the data analysis).	Group allocation was known during treatment administration.<br>Outcome assessments and data analysis were performed without knowledge of group allocation whenever possible.
**Outcome measures**	6	a. Clearly define all outcome measures assessed (e.g. cell death, molecular markers, or behavioural changes).	
		b. For hypothesis-testing studies, specify the primary outcome measure, i.e. the outcome measure that was used to determine the sample size.	The primary outcome measure was the improvement of UVB-induced skin damage as assessed by histological changes and oxidative stress–related biomarkers.
**Statistical methods**	7	a. Provide details of the statistical methods used for each analysis, including software used.	Data are prese [illegible]<br>Statistical analy [illegible]
		b. Describe any methods used to assess whether the data met the assumptions of the statistical approach, and what was done if the assumptions were not met.	Student's t-test was used for comparisons between two groups, and one-way analysis of variance (ANOVA) was used for comparisons among multiple groups.<br>A P value < 0.05 was considered statistically [illegible]
**Experimental animals**	8	a. Provide species-appropriate details of the animals used, including species, strain and substrain, sex, age or developmental stage, and, if relevant, weight.	Female Kunmi [illegible]
		b. Provide further relevant information on the provenance of animals, health/immune status, genetic modification status, genotype, and any previous procedures.	Mice were housed in a specific pathogen-free (SPF) animal facility with free access to food and water. All animals were healthy and had not undergone any previous experimental procedures.
**Experimental procedures**	9	For each experimental group, including controls, describe the procedures in enough detail to allow others to replicate them, including:	
		a. What was done, how it was done and what was used.	A UVB-induced skin photoaging model was established by repeated dorsal UVB irradiation. Topical formulations (Vc, DPP, DPP-LIP, LIP-SA, and DPP-LIP-SA) were [illegible]
		b. When and how often.	UVB irradiation was performed once every two days following a stepwise exposure schedule. Topical formulations were applied 30 minutes before each irradiation session. [illegible]
		c. Where (including detail of any acclimatisation periods).	All procedures were conducted in a specific pathogen-free (SPF) animal facility.<br>Mice were housed under standard laboratory conditions with free access to food and water [illegible]
		d. Why (provide rationale for procedures).	UVB irradiation was used to induce skin photoaging because it mimics UV-induced skin damage in humans.<br>Topical administration was chosen to [illegible]
**Results**	10	For each experiment conducted, including independent replications, report:	
		a. Summary/descriptive statistics for each experimental group, with a measure of variability where applicable (e.g. mean and SD, or median and range).	Results were r [illegible]
		b. If applicable, the effect size with a confidence interval.	Effect sizes and corresponding confidence intervals were not formally calculated.

The Recommended Set

These items complement the Essential 10 and add important context to the study. Reporting the items in both sets represents best practice.

Item		Recommendation	Section/line number, or reason for not reporting
**Abstract**	11	Provide an accurate summary of the research objectives, animal species, strain and sex, key methods, principal findings, and study conclusions.	Skin phot
**Background**	12	a. Include sufficient scientific background to understand the rationale and context for the study, and explain the experimental approach.<br>b. Explain how the animal species and model used address the scientific objectives and, where appropriate, the relevance to human biology.	Skin photoaging is primarily induced by chronic UV radiation and is characterized by oxidative stress, inflammation, and extracellular matrix degradation. Existing topical antioxidants and anti-aging agents are limited by short biological half-lives,<br>A UVB-induced skin photoaging mouse model was used because it reproduces key pathological features of human photoaging, including oxidative stress, inflammatory responses, and collagen degradation, allowing evaluation of dermal protection and repair in
**Objectives**	13	Clearly describe the research question, research objectives and, where appropriate, specific hypotheses being tested.	The objective of this study was to develop a lip
**Ethical statement**	14	Provide the name of the ethical review committee or equivalent that has approved the use of animals in this study, and any relevant licence or protocol numbers (if applicable). If ethical approval was not sought or granted, provide a justification.	All animal experiments were approved by the Committee on Animal Research and Ethics of the School of Pharmacy, Jilin University (Approval No. 20240100), and were conducted in accordance with the NIH Guide for the Care and Use of Laboratory Animals. This study was reported in compliance with the ARRIVE (Animal Research: Reporting of In Vivo Experiments) guidelines.
**Housing and husbandry**	15	Provide details of housing and husbandry conditions, including any environmental enrichment.	Animals were housed in a specific pathogen-fr
**Animal care and monitoring**	16	a. Describe any interventions or steps taken in the experimental protocols to reduce pain, suffering and distress.<br>b. Report any expected or unexpected adverse events.<br>c. Describe the humane endpoints established for the study, the signs that were monitored and the frequency of monitoring. If the study did not have humane endpoints, state this.	Animals were monitored regularly throughout the experimental period.<br>Anesthesia with isoflurane was used prior to euthanasia to minimize pain and distress.<br>No unexpected adverse events occurred during the study.
**Interpretation/ scientific implications**	17	a. Interpret the results, taking into account the study objectives and hypotheses, current theory and other relevant studies in the literature.<br>b. Comment on the study limitations including potential sources of bias, limitations of the animal model, and imprecision associated with the results.	The results demonstrate that incorporation of DPP-loaded liposomes into a sodium alginate hydrogel significantly enhances dermal bioavailability and photoprotective efficacy.<br>The composite system effectively mitigated oxidative stress, suppressed inflammatory responses, and promoted extracellular matrix remodeling in photoaged skin, supporting its potential as a peptide-based topical strategy for skin photoaging intervention.
**Generalisability/ translation**	18	Comment on whether, and how, the findings of this study are likely to generalise to other species or experimental conditions, including any relevance to human biology (where appropriate).	Although this study was conducted in a mouse model, the pathological mechanisms targeted—oxidative stress, inflammation, and collagen degradation—are conserved in human skin photoaging. These findings suggest potential translational relevance for the development of topical anti-photoaging formulations, while further studies in human skin models or clinical settings are warranted.
**Protocol registration**	19	Provide a statement indicating whether a protocol (including the research question, key design features, and analysis plan) was prepared before the study, and if and where this protocol was registered.	No protocol was registered prior to the study
**Data access**	20	Provide a statement describing if and where study data are available.	Data are available from the corresponding auth
**Declaration of interests**	21	a. Declare any potential conflicts of interest, including financial and non-financial. If none exist, this should be stated.<br>b. List all funding sources (including grant identifier) and the role of the funder(s) in the design, analysis and reporting of the study.	The authors declare no conflicts of interest.<br>This work was supported by the Science and Technology Research Project of the Education Department of Jilin Province (No. JJKH20250217KJ).

药学院动物实验伦理审查表
The Tab of Animal Experimental Ethical Inspection

编号(Number of permit)：20240100

申请人填写的相关信息 (Concerned information wrote by applicant)

申请科室(Name of organization)： Department of Biopharmacy

申请人学历(Education of applicant)：MD/PhD　技术职称(Professional title)：Professor　专业(Major)：Pharmaceutics

实验名称(Study title)：A liposome–hydrogel composite ameliorates UVB-induced mouse skin photoaging through integrated antioxidant and extracellular matrix remodeling pathways

实验目的(Aim of experiment)：To evaluate the protective effects and underlying mechanisms of DPP-LIP-SA in a UVB-induced mouse model of skin photoaging.

拟进动物情况

动物来源(Source of animal)：Changsheng Biotechnology Co., Ltd. (Liaoning, China)
Animal license number: NO.SCXK(LIAO)2020---0001

品种品系(Species or strain)：Female Kunming mice　等级（Grade)：SPF grade　规格（Age)：6–8 weeks

数量(Number)：56 mice (n = 8 per group, 7 groups)	申请日期(Application date)：May 1, 2024
进驻日期(Entering date)：August 12, 2024	结束日期(Ending date)：February 1, 2025

实验要点,包括实验方法、观测指标、实验结束后处死动物的方法等(Outline of experiments, experimental methods, observational index, executing animal method et. al)：

1. Outline of experiments. A UVB-induced skin photoaging mouse model was established to evaluate the protective effects and underlying mechanisms of DPP-LIP-SA following topical administration. Mice were exposed to dorsal UVB irradiation according to a stepwise schedule and treated with different formulations prior to each irradiation. Skin morphology, histopathology, oxidative stress, inflammatory responses, and extracellular matrix–related markers were systematically assessed.

2. Experimental methods. Female Kunming mice were exposed to dorsal UVB irradiation (311 nm) once every two days for 8 weeks following a stepwise schedule (10–45 min, 40 mJ/cm²). The dorsal skin area (2 cm × 2 cm) was depilated regularly. Mice were randomly assigned into seven groups (n = 8): Normal, Model, Vc, DPP, DPP-LIP, LIP-SA, and DPP-LIP-SA. For treatment groups, formulations were topically administered at a dose of 1.0 mg active ingredient per cm², 30 min before each irradiation session.

3. Observational index. (i) Skin surface features and skin injury scores; (ii) Body weight and organ coefficients; (iii) Skin moisture content; (iv) Histopathological changes assessed by H&E and Masson staining; (v) Oxidative stress–related biomarkers including SOD, CAT, GSH-Px, MDA, and ROS; (vi) Inflammatory cytokines including IL-1β, IL-6, and TNF-α; (vii) Protein expression of collagen I and MMP-3 by Western blot; (viii) mRNA expression of collagen I, MMP-3, TGF-β1, p53, and SOD2 by RT-qPCR.

4. Executing animal method. At the end of the experiment, mice were anesthetized with isoflurane and euthanized. Retro-orbital blood, full-thickness dorsal skin, and major organs (heart, liver, spleen, lungs, and kidneys) were collected for subsequent histological and biochemical analyses.

5. Ethical consideration. All animal experiments were conducted in accordance with relevant guidelines for the care and use of laboratory animals, and efforts were made to minimize animal suffering and reduce the number of animals used.

申请人签名(Signature of applicant)：[illegible]　联系电话(Telephone)：13843171367

<table>
<tr>
<td>审查
依据
(Inspection contents)</td>
<td colspan="5">1. 该项目是否必须用实验动物进行实验，即能否用计算机模拟、细胞培养等非生命方法替代动物或用低等动物替代高等动物进行实验？(Is this project required to use experimental animals? Can non-living methods such as computer simulation or cell culture, or the use of lower animals, replace the use of experimental animals?)
This project cannot be replaced by non-animal methods and therefore requires the use of experimental animals.
2. 表中所填申请人资格和所用动物的品种品系、质量等级、规格是否合适，能否通过改良设计方案或用高质量的动物来减少所用动物的数量？(Are the applicant's qualifications and the selected animal species/strain, quality grade, and specifications appropriate? Can the number of animals used be reduced by improving the experimental design or using higher-quality animals?)
The applicant's qualifications and the selected animal species/strain, quality grade, and specifications are appropriate, and the number of animals used has been minimized.
3. 能否通过改进实验方法、调整实验观测指标、改良处死动物的方法，来优化实验方案、善待动物？(Can the experimental design be optimized to improve animal welfare by refining experimental methods, adjusting observational indices, or improving the method of euthanasia?)
The experimental procedures were optimized, and animals were euthanized under anesthesia to minimize pain and distress.</td>
</tr>
<tr>
<td rowspan="2">审查结果
(是否同意申请人的实验方案)
(Results of inspection)</td>
<td>委员会专家意见
(Study director):</td>
<td>同意
(Agree) ☑</td>
<td>不同意
(Disagree) ☐</td>
<td colspan="2">签　名
(Signature)</td>
</tr>
<tr>
<td>吉林大学药学院实验动物伦理委员会(Institutional Animal Care and Use Committee of Jilin University School of Pharmaceutical Science):</td>
<td>同意
(Agree) ☑</td>
<td>不同意
(Disagree) ☐</td>
<td colspan="2" rowspan="2">签章(Stamp)</td>
</tr>
<tr>
<td colspan="4">备注(Supplement):</td>
</tr>
</table>
