# Supplementary material for: A liposome–hydrogel composite ameliorates UVB-induced mouse skin photoaging through integrated antioxidant and extracellular matrix remodeling pathways
Source: Drug Deliv. 2026 Jul 30;33(1):2708405. doi: 10.1080/10717544.2026.2708405 (PMC13425532; doi:10.1080/10717544.2026.2708405)
Supplement: Supplementary Material.docx [file IDRD_A_2708405_SM1891.docx]

**Supplementary Information**

**A liposome–hydrogel composite ameliorates UVB-induced mouse skin photoaging through integrated antioxidant and extracellular matrix remodeling pathways**

Yifan Liu *^a b^*, Kejia Chen *^b^*, Jin Pei *^b^*, Qi Song ^a^, Xinlu Wang ^a^, Chuhan Ma *^b^*, Yao Li *^b^*, Liangping Yu *^a^*

*^a^ Department of Clinical Pharmacy, The First Hospital of Jilin University, Changchun, People's Republic of China.*

*^b^ Department of Biopharmacy, School of Pharmaceutical Sciences, Jilin University, Changchun, People's Republic of China.*

^*^ Corresponding author:

Liangping Yu, Department of Clinical Pharmacy, the First Hospital of Jilin University, No. 1 Xinmin Street, Changchun, 130061, People’s Republic of China, Tel +86 431 81879802, Email liangpingyu@jlu.edu.cn

# Supplementary Methods

**Optimization of the DPP-loaded liposomes (DPP-LIP) formulation**

The formulation of DPP-LIP was optimized using a combination of single-factor screening and orthogonal design, with encapsulation efficiency (EE) as the primary response variable. Single-factor experiments were first conducted to evaluate the effects of the phospholipid-to-cholesterol ratio (PC:CHOL), drug-to-lipid ratio (D:L), rotary evaporation temperature, and hydration temperature on EE. Specifically, PC:CHOL ratios ranging from 1:1 to 9:1, D:L ratios from 1:1 to 1:20, evaporation temperatures from 35–55 °C, and hydration temperatures from 25–50 °C were systematically examined using the thin-film hydration method. The corresponding EE values are presented in Supplementary Fig. S2A–D. These experiments revealed clear parameter-dependent trends: EE increased with the PC:CHOL ratio up to 6:1 before declining at higher ratios, and similarly peaked at a D:L ratio of 1:15. Hydration temperature exhibited an optimum at 37 °C, whereas rotary evaporation temperature had comparatively minor influence.

Based on the single-factor results, four variables with the greatest impact were incorporated into a four-factor, three-level orthogonal design (L9(3⁴)) to analyze interaction effects and identify the optimal formulation (Supplementary Tables S1–S3). Range analysis (R-values) together demonstrated that the drug-to-lipid ratio had the strongest influence on EE, followed by the PC:CHOL ratio, hydration temperature, and evaporation temperature. Consistent with the single-factor trends, the optimal combination predicted by the orthogonal design was PC:CHOL = 6:1, D:L = 1:15, evaporation temperature = 45 °C, and hydration temperature = 37 °C. Factor-effect trends derived from K-values are presented in Supplementary Fig. S3.

To verify the reliability of this optimized combination, three independent batches of DPP-LIP were prepared using the selected conditions. The EE values (mean ± SD) demonstrated excellent reproducibility (Supplementary Table S4), confirming the stability and consistency of the optimized formulation.

# Supplementary Tables

**Supplementary Table S1. Formulation factors and levels used in the L9(3^4^) orthogonal design for deer placental peptide-loaded liposomes (DPP-LIP) optimization.**

| Factor | Code | Level 1 | Level 2 | Level 3 |
| --- | --- | --- | --- | --- |
| Phospholipid-to-cholesterol ratio (w/w) | A | 4:1 | 6:1 | 8:1 |
| Drug-to-lipid ratio (w/w) | B | 1:10 | 1:15 | 1:20 |
| Rotary evaporation temperature (°C) | C | 40 | 45 | 50 |
| Hydration temperature (°C) | D | 30 | 37 | 45 |

**Supplementary Table S2. L9(3^4^) orthogonal array and encapsulation efficiency (EE) results for deer placental peptide-loaded liposomes (DPP-LIP) (n = 3).**

| Run | A (PC:CHOL) | B (D:L) | C (°C) | D (°C) | EE (%) |
| --- | --- | --- | --- | --- | --- |
| 1 | 4:1 | 1:10 | 40 | 30 | 62.67 |
| 2 | 4:1 | 1:15 | 45 | 37 | 71.38 |
| 3 | 4:1 | 1:20 | 50 | 45 | 60.32 |
| 4 | 6:1 | 1:10 | 45 | 45 | 71.74 |
| 5 | 6:1 | 1:15 | 50 | 30 | 76.65 |
| 6 | 6:1 | 1:20 | 40 | 37 | 68.69 |
| 7 | 8:1 | 1:10 | 50 | 37 | 67.73 |
| 8 | 8:1 | 1:15 | 40 | 45 | 72.68 |
| 9 | 8:1 | 1:20 | 45 | 30 | 63.11 |

**Note: (A)** Phospholipid-to-cholesterol ratio (w/w); **(B)** Drug-to-lipid ratio (w/w); **(C)** Rotary evaporation temperature (°C); **(D)** Hydration temperature (°C).

**Supplementary Table S3. Level means (K-values) and range values (R-values) derived from the L9(3⁴) orthogonal design.**

| Factor | K1 | K2 | K3 | R |
| --- | --- | --- | --- | --- |
| A | 64.79 | 72.36 | 67.84 | 7.57 |
| B | 67.38 | 73.57 | 64.04 | 9.53 |
| C | 68.01 | 68.74 | 68.23 | 0.73 |
| D | 67.48 | 69.27 | 68.25 | 1.79 |

**Note: (A)** Phospholipid-to-cholesterol ratio (w/w); **(B)** Drug-to-lipid ratio (w/w); **(C)** Rotary evaporation temperature (°C); **(D)** Hydration temperature (°C).

**Supplementary Table S4. Verification of the optimized deer placental peptide-loaded liposomes (DPP-LIP) formulation (n = 3).**

|  | Batch 1 | Batch 2 | Batch 3 | Mean ± SD |
| --- | --- | --- | --- | --- |
| EE (%) | 78.24 | 76.36 | 77.74 | 77.45 ± 0.97 |

**Supplementary Table S5. Skin scoring criteria for UVB-induced photoaging in mice.**

| Score | Detailed description |
| --- | --- |
| 0 | Smooth skin with no visible wrinkles, erythema, or dryness. |
| 1 | Slight fine lines or surface roughness. No erythema or visible thickening. |
| 2 | Mild visible fine wrinkles with early signs of skin coarseness or dullness. No deep folds. |
| 3 | Multiple shallow wrinkles with moderate skin roughness and occasional mild erythema. |
| 4 | Prominent coarse wrinkles with obvious roughness, slight sagging, and intermittent erythema or flaking. |
| 5 | Deep, widespread wrinkles with marked skin thickening, severe dryness/roughness, redness, and scaling. |

**Supplementary Table S6. Primer sequences used for RT-qPCR.**

| Gene | Forward primer (5′→3′) | Reverse primer (5′→3′) |
| --- | --- | --- |
| Collagen I | CTGGAGGAGGACACAGAG | GAGGGAGGTGAGAGGAAGAC |
| Collagen III | CTCAGGGTGTCAAGGGTGAAAGTG | TGTACCAGCCAGACCAGGAAGAC |
| MMP-3 | TGTTCTTCAGAGAGGAGCAG | GTTGGAGTGGAGTGAGGAC |
| TGF-β1 | AGTGACCAGGGTGAACTGAG | GAGGGAAGGCCGTGATGAC |
| p53 | GAGGAGCGGAGGAGTTTG | CGGGTGGTAGTTAGGGTG |
| SOD2 | AGCCTGAAGGAGTTCAGGA | CTTCACCCAAACGATGACAG |
| GAPDH | GAAGGTGAAGGTCGGAGTC | GAAGATGGTGATGGGATTTC |

# Supplementary Figures


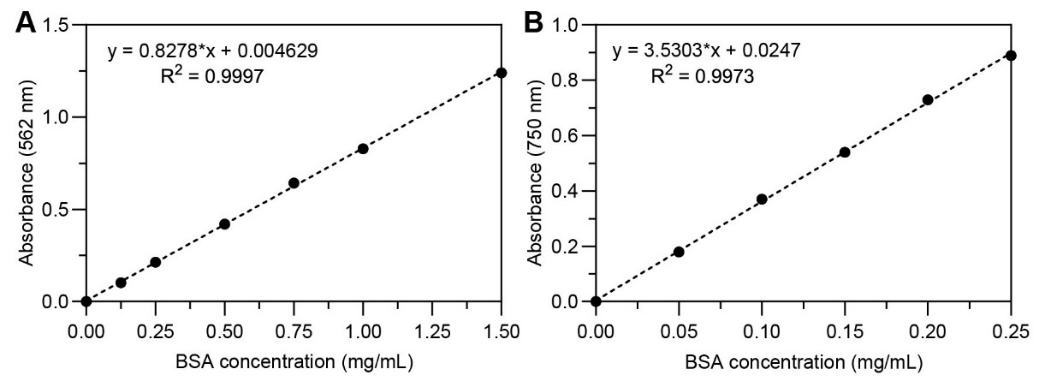


**Supplementary Figure S1. Calibration curves for protein and peptide quantification.**

**(A)** BCA calibration curve for DPTP quantification using BSA standard (562 nm). **(B)** Folin–Ciocalteu calibration curve for DPP quantification using tyrosine standard (750 nm).


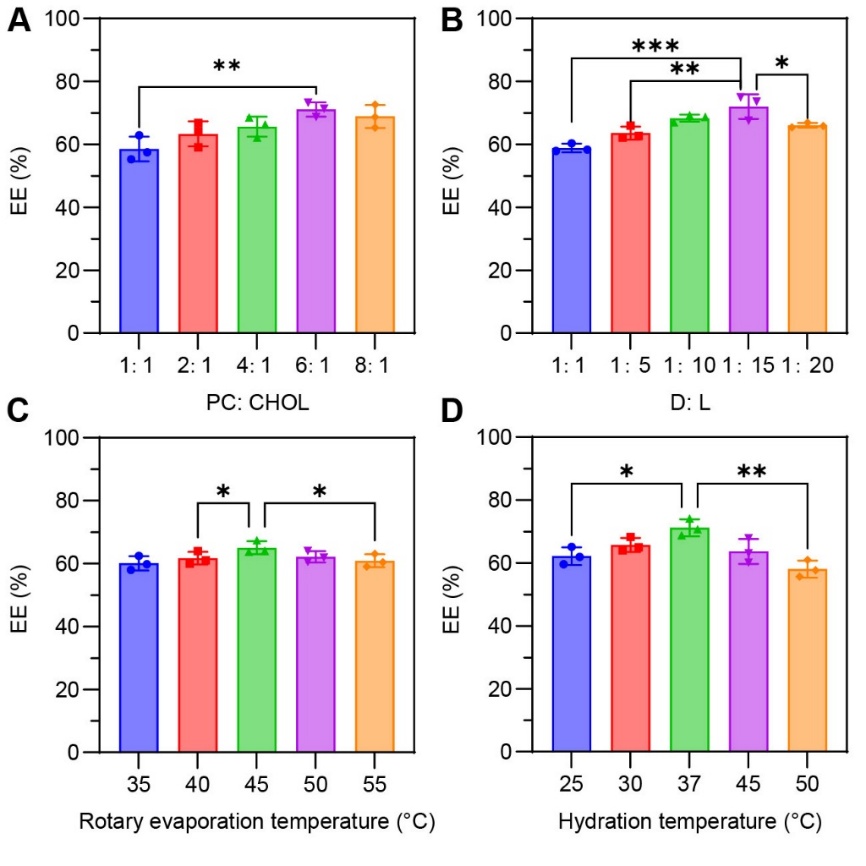


**Supplementary Figure S2. Effects of formulation variables on the encapsulation efficiency (EE) of deer placental peptide-loaded liposomes (DPP-LIP).**

**(A)** Effect of phospholipid-to-cholesterol ratio (PC:CHOL = 1:1, 2:1, 4:1, 6:1, 9:1) on EE (%). **(B)** Effect of drug-to-lipid ratio (D:L = 1:1, 1:5, 1:10, 1:15, 1:20, w/w) on EE (%). **(C)** Effect of rotary evaporation temperature (35, 40, 45, 50, 55 °C) on EE (%). **(D)** Effect of hydration temperature (25, 30, 37, 45, 50 °C) on EE (%). Histogram data are presented as the mean ± SD, n = 3; ^*^*P* < 0.05, ^**^*P* < 0.01,and ^***^*P* < 0.001.


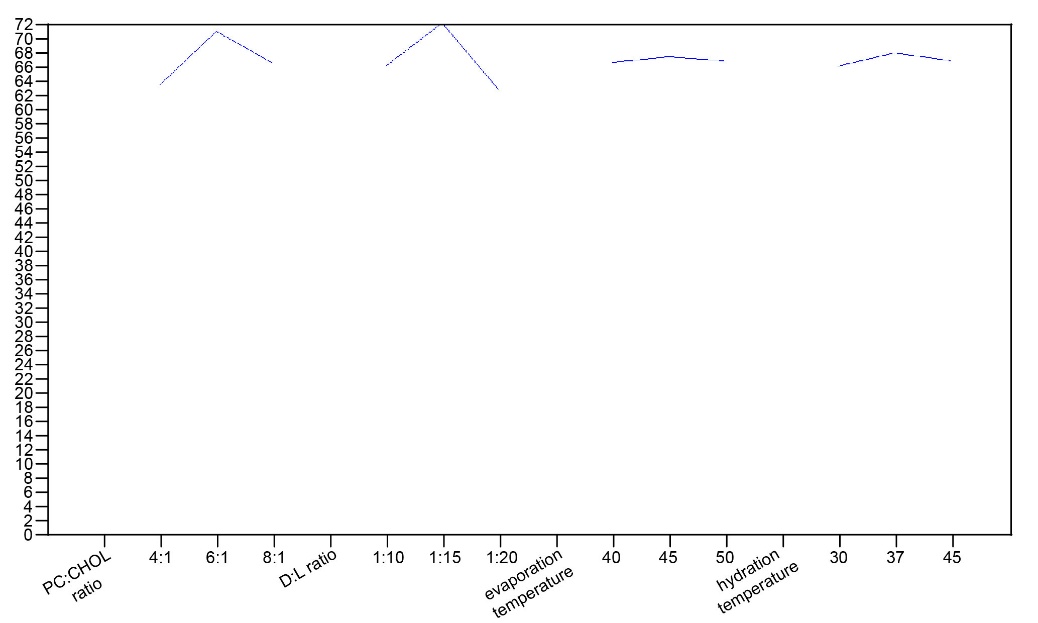


**Supplementary Figure S3. Factor-effect trend plots from the L9(3^4^) orthogonal design.**

Effect of each formulation factor (PC:CHOL ratio, D:L ratio, evaporation temperature, and hydration temperature) on EE% based on K-values from orthogonal analysis.


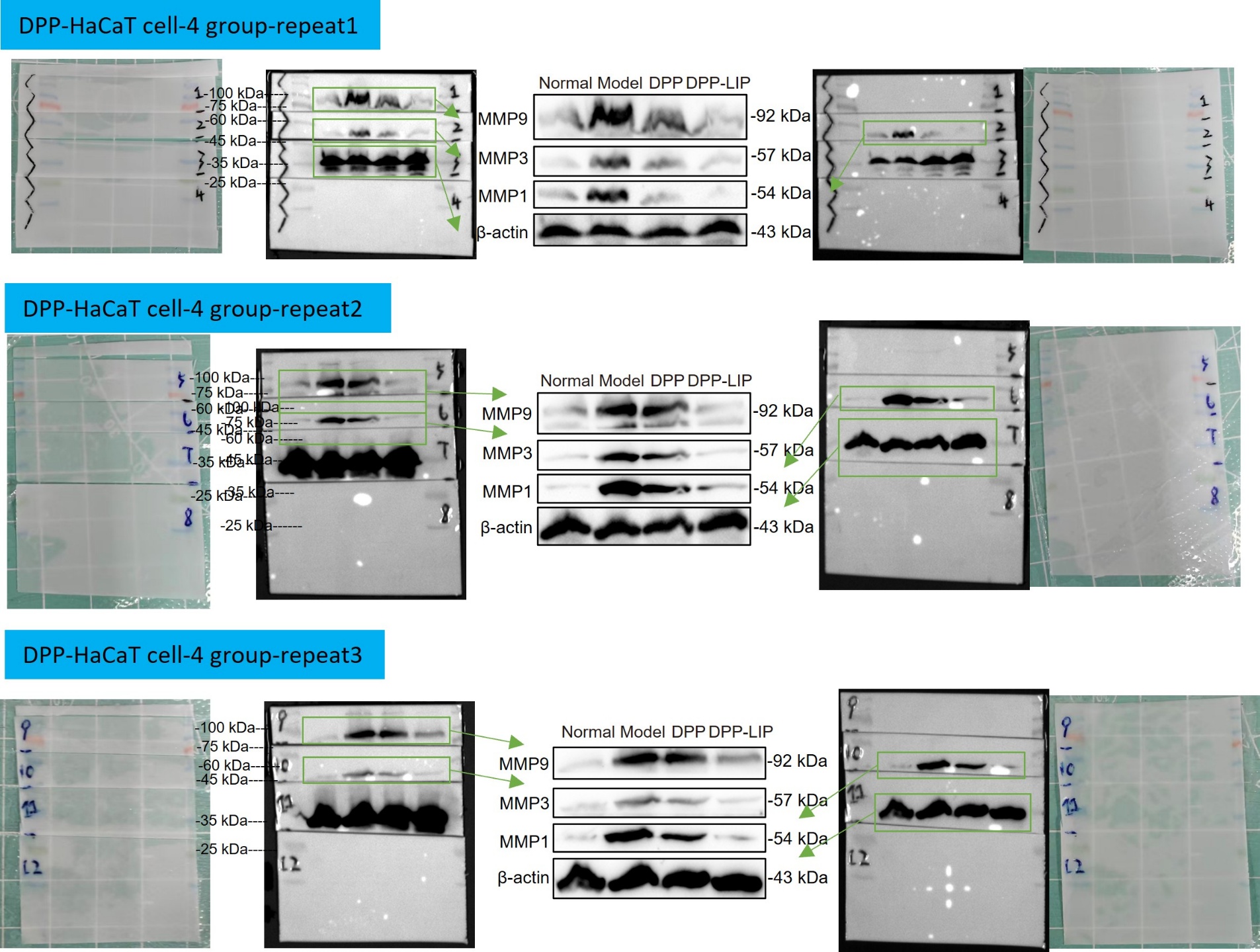


**Supplementary Figure S4. Original western blot images from three independent experiments corresponding to Figure 3M.**


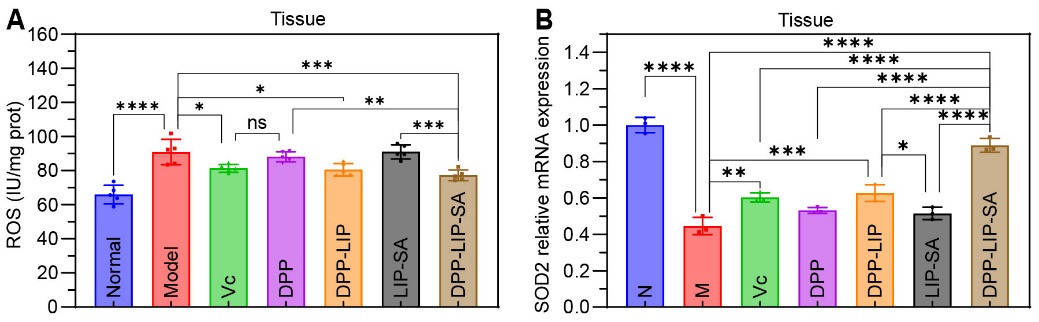


**Supplementary Figure S5.** **Analyses of ROS and SOD2 in skin tissues.**

**(A)** ELISA analysis of ROS levels in skin tissues. **(B)** RT-qPCR analysis of SOD2 mRNA expression in skin tissues. Data are presented as the mean ± SD (n = 5). *P < 0.05, **P < 0.01, ***P < 0.001, and ****P < 0.0001.

**
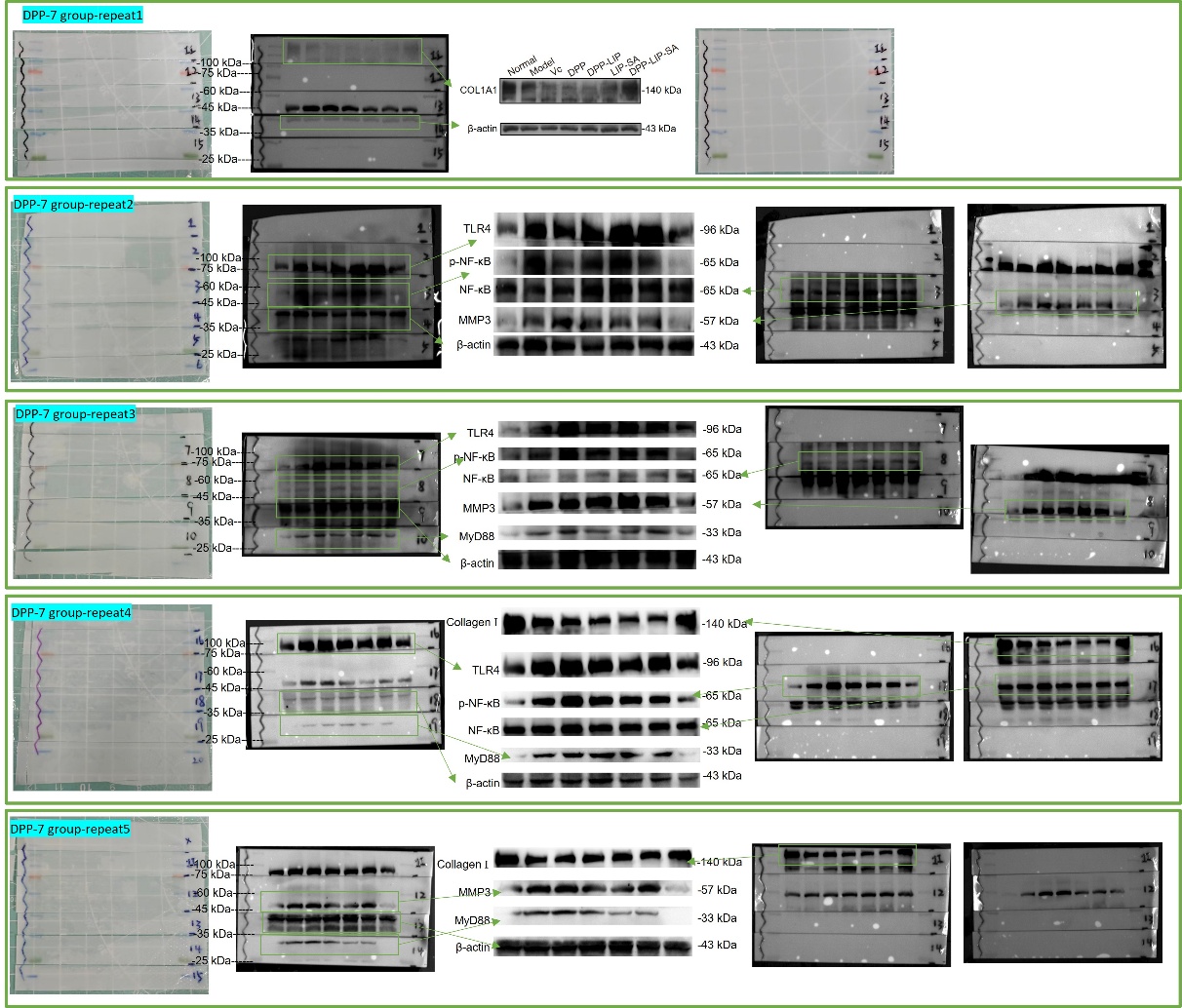
**

**Supplementary Figure S6. Original uncropped western blot images from independent experiments corresponding to Figure 8D and 9D.**
